# Association of saturated fatty acids with cancer risk: a systematic review and meta-analysis

**DOI:** 10.1186/s12944-024-02025-z

**Published:** 2024-01-30

**Authors:** Jin Mei, Meiyu Qian, Yanting Hou, Maodi Liang, Yao Chen, Cuizhe Wang, Jun Zhang

**Affiliations:** Medical College of Shihezi University, Bei-Er-Lu, Shihezi, Xinjiang 832000 China

**Keywords:** Saturated fatty acids, Cancer susceptibility, Primary prevention, Meta-analysis, Systematic review

## Abstract

**Objective:**

Extensive research has explored the link between saturated fatty acids (SFAs) and cardiovascular diseases, alongside other biological dysfunctions. Yet, their association with cancer risk remains a topic of debate among scholars. The present study aimed to elucidate this association through a robust meta-analysis.

**Methods:**

PubMed, Embase, Cochrane Library, and Web of Science databases were searched systematically to identify relevant studies published until December 2023. The Newcastle-Ottawa Scale was used as the primary metric for evaluating the quality of the included studies. Further, fixed- or random-effects models were adopted to determine the ORs and the associated confidence intervals using the Stata15.1 software. The subsequent subgroup analysis revealed the source of detection and the cancer types, accompanied by sensitivity analyses and publication bias evaluations.

**Results:**

The meta-analysis incorporated 55 studies, comprising 38 case-control studies and 17 cohort studies. It revealed a significant positive correlation between elevated levels of total SFAs and the cancer risk (OR of 1.294; 95% CI: 1.182–1.416; *P-*value less than 0.001). Moreover, elevated levels of C14:0, C16:0, and C18:0 were implicated in the augmentation of the risk of cancer. However, no statistically significant correlation of the risk of cancer was observed with the elevated levels of C4:0, C6:0, C8:0, C10:0, C12:0, C15:0, C17:0, C20:0, C22:0, and C24:0. Subgroup analysis showed a significant relationship between excessive dietary SFA intake, elevated blood SFA levels, and heightened cancer risk. Increased total SFA levels correlated with higher risks of breast, prostate, and colorectal cancers, but not with lung, pancreatic, ovarian, or stomach cancers.

**Conclusion:**

High total SFA levels were correlated with an increased cancer risk, particularly affecting breast, prostate, and colorectal cancers. Higher levels of specific SFA subtypes (C14:0, C16:0, and C18:0) are also linked to an increased cancer risk. The findings of the present study would assist in providing dietary recommendations for cancer prevention, thereby contributing to the development of potential strategies for clinical trials in which diet-related interventions would be used in combination with immunotherapy to alter the levels of SFAs in patients and thereby improve the outcomes in cancer patients. Nonetheless, further high-quality studies are warranted to confirm these associations.

**Supplementary Information:**

The online version contains supplementary material available at 10.1186/s12944-024-02025-z.

## Introduction

Globally, cancer is second to ischemic heart disease as the leading death cause. In 2020, the global incidence of cancer in men was reported at 222.0 per 100,000, with a mortality rate of 120.8 per 100,000 individuals, while the incidence rate in women was 186.0 per 100,000 individuals, with a mortality rate of 84.2 per 100,000 individuals. Forecasts predict a marked escalation in cancer incidence and mortality rates in the coming decades, with expectations of reaching 28.4 million cases worldwide by 2040. Cancer has been predicted to potentially become the leading cause of death by the 2060s [1, 2]. Various risk factors for cancer have been identified to date, a few of which reportedly exert reversible effects on tumorigenesis. Therefore, global cooperation is necessary to clarify more risk factors for cancer and their specific molecular mechanisms related to tumorigenesis to establish a theoretical foundation for further enhancing the prevention, screening, and control of cancer.

Cancer is a complex disease arising due to interactions between genetic and environmental factors. The prevalent environmental risk factors for cancer include smoking, radiation, and alcohol consumption, which contribute significantly to tumor onset. Moreover, with increasing urbanization and an upswing in living standards, inappropriate dietary practices are being increasingly recognized as significant risk factors for tumor development. The ever-increasing preference for fried food, red meat, and smoked food has led to nutritional imbalances in individuals, which has significantly jeopardized the levels of health in the population. SFAs are formed of carbon chains that do not contain unsaturated double bonds and represent an important group of risk factors for cancer. SFAs are the primary influencers of plasma cholesterol levels and are, therefore, extensively studied in the context of cardiovascular health. Certain subtypes of SFAs have been demonstrated to play biological roles in the promotion of inflammation [3–8]. However, the correlation between SFAs and the risk of cancer has been explored less, and no conclusions have been reached so far. While a few studies suggest a definite connection between SFAs and an increased risk of cancer [9, 10], certain others reported a potential anticancer effect of SFAs [11, 12]. The underlying mechanisms have also not been elucidated to date. Consequently, the WCRF/AICR has categorized the relationship between SFA intake and cancer risk as “Limited–no conclusion” [13].

The existing literature does not contain any systematic reviews of studies on the association between SFA levels and cancer risk. This study, therefore, seeks to fill this gap by conducting a systematic review of existing literature to shed light on the potential connection between saturated fatty acid content and cancer risk.

## Methods

This study adheres to the PRISMA guidelines (Supplementary File 1). The systematic review registration is available under the PROSPERO registration no. **CRD42023420444**. Table 1 lists the PICOS criteria used in the present study.

**Table 1:** Application of PICOS criteria for study inclusion

Parameter	Criteria
Participants	Inclusion criteria: 1) ≥ 18 years adults 2) With or without cancer
	Exclusion criteria: 1) Have any mental disorders 2) Whose blood sample cannot be got for any reason, etc.
Intervention/exposure	Patients with cancer or patients with high level SFA
Comparison	Patients without cancer or patients with low level SFA
Outcome	A dichotomous outcome: cancer or no cancer diagnosis. (can be overall or site-specific cancer)
Study design	Observational study, mainly case-control and cohort. Only human studies were considered, no animal studies

### Search strategy

The study involved searching for relevant literature published until December 6, 2023, in multiple databases (PubMed, Embase, Cochrane Library, and Web of Science) using the search terms “saturated fatty acids”, “cancer”, and “observational study”. Both MeSH terms and free-text terms were used in the PubMed, Embase, and Cochrane Library database search, and MeSH terms were searched for in the PubMed search, while the free-text terms were obtained from the ones listed in the PubMed and Embase databases. No restrictions were applied on the country or language during the search process. Synonyms, near-synonyms, and related terms were expanded appropriately to facilitate comprehensive literature retrieval. The search process was primarily computer-aided and supplemented with manual searching and reference tracking to collect relevant studies as comprehensively as possible (Supplementary File 2).

### Eligibility criteria

All retrieved articles were analyzed using EndNote X9. After the removal of duplicates, the residual articles underwent a two-stage screening process by two independent researchers. The initial screening involved reviewing titles and abstracts. Studies that aligned with the preliminary criteria were then subjected to a comprehensive review of the full texts. In the event of disagreement, the two researchers would re-read the full texts and discuss them with a third author. The inclusion criteria were: (1) studies were case-control, cohort, or cross-sectional in nature; (2) depending on the study type, the subjects were divided into a cancer group/non-cancer group or classified into ≥ 2 groups based on the SFA levels. The exclusion criteria were: (1) meta-analyses, reviews, conference materials, conference abstracts, case reports, letters, guidelines, etc.; (2) animal experiment studies; (3) unavailability of full texts.

### Information gathering and rigorous quality appraisal of studies

Two researchers conducted the literature searches and screenings independently, reviewing the potentially relevant articles and extracting essential information that was later tabulated in a predefined format. The extracted information included the authors, the publication year, country origin, study type, cancer type, sample size, age, the type of SFAs, the source of detection, the adjusted OR, RR, HR, 95% CIs, and the adjusting factors.

The methodological integrity of each study was rigorously evaluated employing the NOS [14]. This evaluation process distinctively segregated case-control from cohort studies. In case-control studies, critical assessment criteria included the selection process of cases and controls, the comparability between groups, and the precision in exposure ascertainment. Cohort studies underwent a similar scrutiny focusing on the selection of cohort groups, group comparability, and the thoroughness of outcome evaluations. The maximum total score in NOS is 9. The quality of each study was, therefore, designated as low (≤ 4), medium (5–6), or high (≥ 7).

### Statistical analysis

Binary variable data were pooled as ORs with 95% CI using the Stata15.1 software. To assess the degree of heterogeneity across the included studies, both the Q test and *I*^*2*^ statistics were employed. For instances where *I*^*2*^ was equal to or exceeded 50% and the *P*-value was less than 0.1, a random-effects model was used. Conversely, when *I*^*2*^ was below 50% and the *P*-value surpassed 0.1, a fixed-effects model was utilized. Significant heterogeneity (*I*^*2*^ > 50%) necessitated a subgroup analysis, stratified by detection source and cancer type. A sensitivity analysis was also conducted by sequentially excluding each article to verify the robustness of the meta-analysis. The potential for publication bias was assessed through both Begg’s and Egger’s tests. In cases where bias was detected, a corrective “trim-and-fill” method was employed. The criterion for statistical significance was set at a two-sided *P*-value below 0.05.

## Results

### Overview of literature search outcomes

**Fig. 1:**
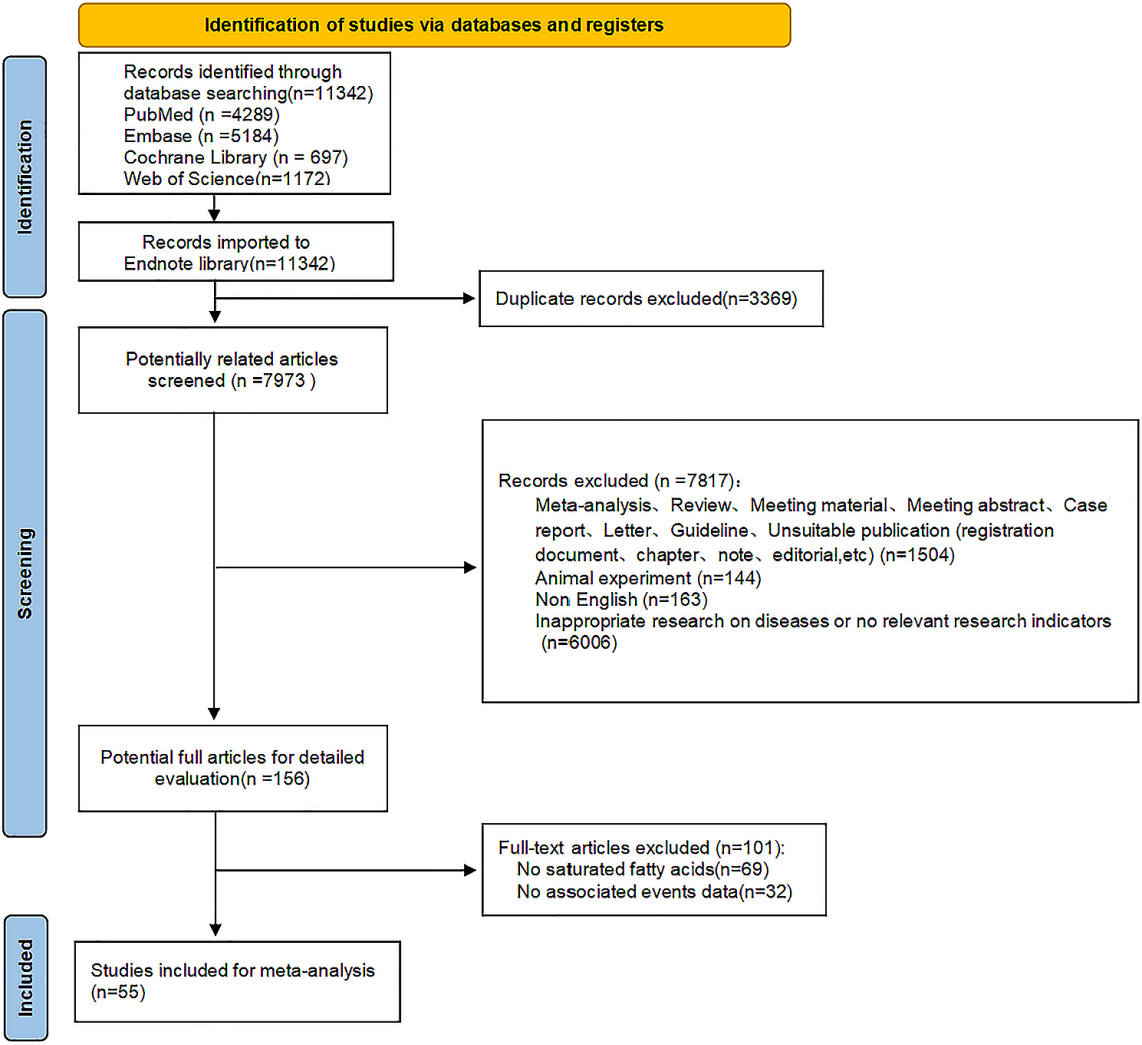
Flowchart of the process of the literature search

Initially,11,342 articles were gathered from various databases, including PubMed, Embase, the Cochrane Library, and Web of Science. After duplicates removal, 7,973 articles remained for analysis. The retained articles were first subjected to an initial screening that involved reading titles and abstracts. In this screening, 156 articles were selected, which were subjected to the next screening step of full-text reading for the exclusion of articles that either lacked information on SFAs or did not provide the necessary data. Finally, 55 articles were retained for the meta-analysis (seen in Fig. 1).

### Study characteristics and quality of the included studies

The studies had been published between the years 1990 and 2023 and comprised 38 case-control studies [15–52] and 17 cohort studies [9, 53–68]. These studies were conducted across different countries, including those from Europe, the United States, Canada, Australia, Iran, China, Japan, and South Korea. Sample sizes ranged from 102 to 525,473 participants, aged 18 to 99 years (Table 2).

**Table 2:** Descriptive data for all 55 included studies

Author, year	Country	Study design	Cancer	SFAs test source	Case		Control/Noncase	
					Numbers (M/F)	Age (y) mean ± SD or min-max	Numbers (M/F)	Age (y) mean ± SD or min-max
Wu et al. (2023) [51]	China	Case-Control	Colorectal	Blood	680 (350/330)	53.69 ± 10.71	680 (344/336)	53.25 ± 10.60
Nkondjock et al. (2003) [15]	Canada	Case-Control	Colorectal	Dietary Intake	402 (200/202)	20–79	668 (239/449)	20–79
Mozafarinia et al. (2021) [16]	Iran	Case-Control	Breast	Dietary Intake	473 (0/473)	45.8 ± 10.3	501 (0/501)	43.9 ± 11.2
Seyyedsalehi et al. (2022) [17]	Iran	Case-Control	Colorectal	Dietary Intake	865	58.5	3206	57.1
Fan et al. (2022) [18]	China	Case-Control	Oral	Dietary Intake	446 (258/188)	≥ 18	448 (213/235)	≥ 18
Tu et al. (2022) [19]	China	Case-Control	Colorectal	Dietary Intake	2806 (1606/1200)	57.11 ± 10.28	2806 (1606/1200)	57.06 ± 9.90
Cai et al. (2020) [53]	Japan	Cohort	Lung	Dietary Intake	1315 (901/414)	45–74	71872 (32033/39839)	45–74
Shimomura et al. (2022) [54]	Japan	Cohort	Non-Hodgkin Lymphoma	Dietary Intake	230	49–64	93136	49–64
Takata et al. (2009) [20]	USA	Case-Control	Breast	Blood	130 (0/130)	58.6 ± 5.1	257 (0/257)	58.6 ± 5.4
Chun et al. (2015) [21]	Korea	Case-Control	Colorectal	Dietary Intake	150 (94/56)	20–79	116 (71/45)	20–79
Jackson et al. (2012) [22]	Jamaica	Case-Control	Prostate	Blood	209 (209/0)	67.5 ± 7.9	226 (226/0)	62.6 ± 10.5
Chavarro et al. (2013) [23]	USA	Case-Control	Prostate	Blood	476 (0/476)	58 ± 8.1793	476 (0/476)	58 ± 7.4358
Nkondjock et al. (2003) [24]	Canada	Case-Control	Breast	Dietary Intake	414	55.0 ± 11.87	429	55.7 ± 12.18
Pan et al. (2004) [25]	Canada	Case-Control	Ovarian	Dietary Intake	442 (0/442)	55.1 ± 12.3	2135 (0/2135)	55.2 ± 12.5
Shishavan et al. (2020) [26]	Iran	Case-Control	Pancreatic	Blood	51 (26/25)	58.59 ± 9.23	152 (79/73)	58.22 ± 9.05
Matta et al. (2022) [27]	USA	Case-Control	Breast	Blood	905	68.2 ± 5.7	1813	68.2 ± 5.7
Matejcic et al. (2018) [28]	Denmark France Greece Germany Italy Netherlands Norway Spain Sweden UK	Case-Control	Pancreatic	Blood	375 (153/222)	64.59 ± 8.87	375 (153/222)	57.44 ± 8.28
Gilsing et al. (2011) [55]	Netherlands	Cohort	Ovarian	Dietary Intake	340	61.8 ± 4.3	2161	61.4 ± 4.3
Kurahashi et al. (2008) [56]	Japan	Cohort	Prostate	Dietary Intake	329	45–74	43106	45–74
Vlajinac et al. (1997) [29]	Serbia	Case-Control	Prostate	Dietary Intake	101 (101/0)	70.5	202 (202/0)	71.5
Hodge et al. (2015) [57]	Italy Greek	Cohort	Colorectal	Dietary Intake Blood	395 (197/198)	61.4 ± 8.1	3810 (1697/2113)	54.3 ± 11.1
Lof et al. (2007) [58]	Sweden	Cohort	Breast	Dietary Intake	974 (0/974)	39 ± 3	43595 (0/43595)	39 ± 3
Knekt et al. (1990) [59]	Finland	Cohort	Breast	Dietary Intake	54 (0/54)	47.2 ± 12.4	3934 (0/3934)	41.1 ± 13.7
Thiébaut et al. (2009) [60]	USA	Cohort	Pancreatic	Dietary Intake	1337 (865/472)	50–71	524136 (307871/216265)	50–71
Kraja et al. (2015) [61]	Netherlands	Cohort	Colorectal	Dietary Intake	222 (97/125)	≥ 55	4754	≥ 55
Aglago et al. (2021) [62]	Denmark France Greece Germany Italy Netherlands Norway Spain Sweden UK	Cohort	Colorectal	Dietary Intake Blood	6162 (43%/57%)	57.1 ± 7.79	443950 (29%/71%)	51.0 ± 9.75
Shishavan et al. (2021) [63]	Iran	Cohort	Pancreatic	Dietary Intake	76 (41/35)	58.11 ± 9.49	46904 (19562/27342)	51.83 ± 8.80
Zhu et al. (2019) [30]	China	Case-Control	Stomach	Dietary Intake	1900 (1401/499)	64.1 ± 10.8	6532(4713/1819)	64.0 ± 11.3
Sczaniecka et al. (2012) [64]	USA	Cohort	Breast	Dietary Intake	772	50–76	29480	50–76
Wakai et al. (2005) [65]	Japan	Cohort	Breast	Dietary Intake	129	40–79	26162	40–79
Shannon et al. (2007) [31]	China	Case-Control	Breast	Blood	330	≥35	1038	≥35
Hirko et al. (2018) [32]	USA	Case-Control	Breast	Blood	794	44.7 (4.5)	794	44.8 (4.4)
Crowe et al. (2008) [33]	Denmark Germany Greece Italy Netherlands Spain Sweden UK	Case-Control	Prostate	Blood	962	60.4 ± 5.8	1061	60.1 ± 5.7
Wakai et al. (2000) [34]	Japan	Case-Control	Bladder	Dietary Intake	300 (243/57)	20–99	300 (243/57)	20–99
Bravi et al. (2013) [35]	Italy Switzerland	Case-Control	Oral and Pharyngeal	Dietary Intake	768 (593/175)	58 (22–79)	2078 (1368/710)	59 (19–79)
Challier et al. (1998) [36]	French	Case-Control	Breast	Dietary Intake	345	30–78	345	30–78 (± 13 years)
Voorrips et al. (2002) [66]	Netherlands	Cohort	Breast	Dietary Intake	941	55–69	1598	55–69
Do et al. (2003) [37]	Korean	Case-Control	Breast	Dietary Intake	224	20–69	250	20–69
Gong et al. (2010) [38]	USA	Case-Control	Pancreatic	Dietary Intake	532 (291/241)	21–85	1701 (883/818)	21–85
Lucenteforte et al. (2008) [39]	Italy	Case-Control	Endometrial	Dietary Intake	454	60 (18–79)	908	61 (19–79)
Lucenteforte et al. (2009) [40]	Italy	Case-Control	Stomach	Dietary Intake	230 (143/87)	63 (22–80)	547 (286/261)	63 (22–80)
Lucenteforte et al. (2010) [41]	Italy	Case-Control	Pancreatic	Dietary Intake	326 (174/152)	63 (34–80)	652 (348/304)	63 (34–80)
Bidoli et al. (2008) [42]	Italy	Case-Control	Renal Cell	Dietary Intake	767 (494/273)	62 (24–79)	1534 (988/546)	62 (22–79)
Jessri et al. (2011) [43]	Iran	Case-Control	Esophageal Cquamous Cell Carcinoma	Dietary Intake	47 (18/29)	40–75	96 (38/58)	40–75
Bidoli et al. (2002) [44]	Iran	Case-Control	Ovarian	Dietary Intake	1031	56 (18–79)	2411	57 (17–79)
Polesel et al. (2007) [45]	Italy	Case-Control	Hepatocellular carcinoma	Dietary Intake	185 (149/36)	66 (43–84)	412 (281/131)	65 (40–82)
Bassett et al. (2013) [46]	Australia	Case-Control	Prostate	Dietary Intake Blood	464	62.7 ± 6.0	1661	54.1 ± 11.4
Pouchieu et al. (2014) [47]	UK	Case-Control	Overall	Blood	250 (80/170)	51.0 ± 6.0	250 (80/170)	51.3 ± 6.2
Wise et al. (2014) [67]	USA	Cohort	Uterine Leiomyomata	Dietary Intake	2695	21–69	9349	21–69
Luu et al. (2018) [68]	China	Cohort	Lung	Dietary Intake	1496	Female: 40–70 Male: 40–74	120474	Female: 40–70 Male: 40–74
Kuriki et al. (2006) [48]	Japan	Case-Control	Colorectal	Blood	74 (45/29)	58.4 ± 9.6	221 (134/87)	58.0 ± 9.6
Sellem et al. (2018) [9]	French	Cohort	Overall	Dietary Intake	1722	56.9 ± 7.4	42317	56.9 ± 7.4
Saadatian-Elahi et al. (2002) [49]	USA	Case-Control	Breast	Blood	197	34–65	197	34–65
Vinceti et al. (2013) [50]	Italy	Case-Control	Cutaneous Melanoma	Blood	51 (23/28)	25–79	51 (23/28)	25–79
Nkondjock et al. (2005) [52]	Canada	Case-Control	Pancreatic	Dietary Intake	462 (258/204)	30–74	4721 (2331/2309)	30–74

Supplementary Table 1 lists the outcome measures (OR, HR, and RR), the interpretation, and the confounders used for all studies. The primary focus of most studies was on total SFAs, and just a few studies also discussed specific SFA subtypes. In 41 studies among all the included ones, SFA intake was measured through a survey using questionnaires as the most common approach. On the other hand, 17 studies adopted the method of direct measurement of the total SFA content in the blood samples.

Further, the association between 15 types of cancer and total SFAs was evaluated across the cohort and case-control studies. The cancers were ranked in descending order based on the number of papers published on them, are: breast cancer (12 studies, 5938 cases), colorectal cancer (8 studies, 11,076 cases), pancreatic cancer (5 studies, 1290 cases), prostate cancer (4 studies, 1370 cases), ovarian cancer (3 studies, 1813 cases), lung cancer (2 studies, 1315 cases), stomach cancer (2 studies, 2130 cases), and single-study representations for bladder cancer (300 cases), endometrial cancer (454 cases), esophageal squamous cell carcinoma (47 cases), hepatocellular carcinoma (185 cases), non-Hodgkin lymphoma (230 cases), oral and pharyngeal cancer (768 cases), oral cancer (446 cases), and renal cell carcinoma (767 cases).

In the assessment of these 55 studies using the NOS, most studies received a score of 7 (*n* = 33) or 8 (*n* = 15), while the remaining studies received a score of 9 (*n* = 8) (seen in Supplementary Table 2).

### Statistical analysis

#### Association between total SFAs and the cancer risk

**Fig. 2:**
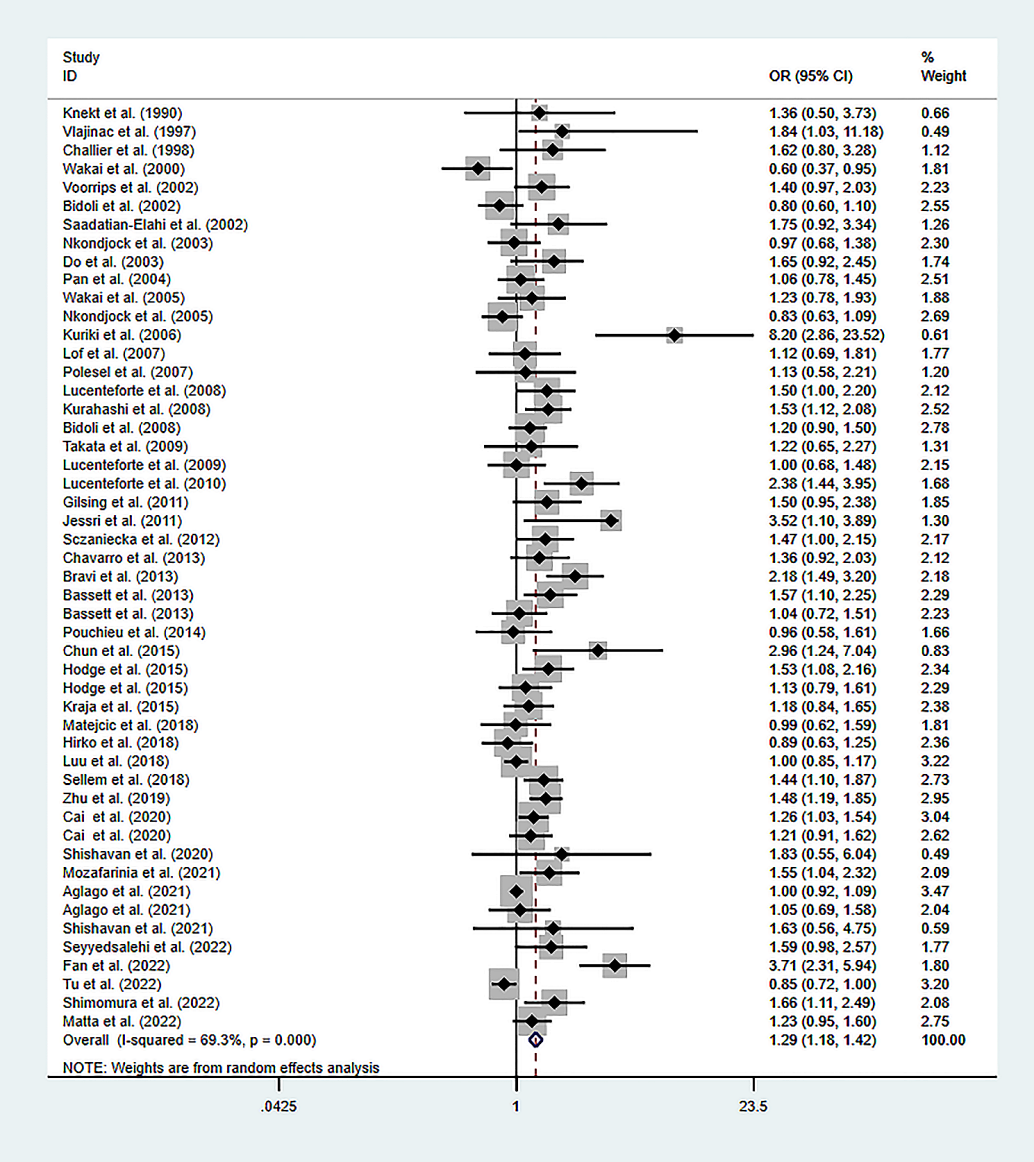
Meta-analysis of the studies on the association of total SFA levels and the risk of cancer

The heterogeneity analysis for total SFAs revealed an *I*^*2*^ of 69.3% and *P*-value less than 0.001. Therefore, a random-effects model was selected. The pooled data indicated a significant positive correlation between high levels of total SFAs and the cancer risk (OR of 1.294; 95% CI: 1.182–1.416; *P*-value less than 0.001), as depicted in Fig. 2.

#### Association between different subtypes of SFAs and the cancer risk

For C14:0, the heterogeneity analysis revealed an *I*^*2*^ of 70.4% and a *P*-value less than 0.001, leading to the selection of a random-effects model. The pooled data indicated a significant positive correlation between high levels of C14:0 and increased cancer risk, with an OR of 1.191 and a 95% CI spanning from 1.050 to 1.351, and a *P*-value of 0.007.

In the case of C15:0, heterogeneity analysis showed an *I*^*2*^ of 0.0% and a *P*-value of 0.738, prompting the use of a fixed-effects model. The pooled data suggested a lack of statistically significant correlation between high levels of C15:0 and cancer risk, with an OR of 1.043 and a 95% CI of 0.964 to 1.144, and a *P*-value of 0.129.

For C16:0, the heterogeneity analysis revealed an *I*^*2*^ of 71.4% and a *P*-value less than 0.001, necessitating the use of a random-effects model. The data showed a significant positive correlation between high levels of C16:0 and cancer risk, with an OR of 1.267 and a 95% CI of 1.131 to 1.420, and a *P*-value less than 0.001.

The analysis for C17:0 indicated an *I*^*2*^ of 79.8% and a *P*-value less than 0.001, leading to the selection of a random-effects model. However, the data showed no significant correlation between high levels of C17:0 and cancer risk, with an OR of 1.048, a 95% CI of 0.813 to 1.351, and a *P*-value of 0.72.

In the case of C18:0, heterogeneity analysis revealed an *I*^*2*^ of 57.5% and a *P*-value less than 0.001, leading to the use of a random-effects model. The data indicated a significant positive correlation between high levels of C18:0 and cancer risk, with an OR of 1.177, a 95% CI of 1.073 to 1.292, and a *P*-value of 0.001.

For other subtypes like C4:0, C6:0, C8:0, C10:0, C12:0, C20:0, C22:0, and C24:0, heterogeneity analysis showed *I*^*2*^ values greater than 50% with *P*-values less than 0.1. A random-effects model was selected for these. The pooled data did not establish a statistically significant linkage between an elevated cancer risk and increased concentrations of certain SFAs, including C4:0, C6:0, C8:0, C10:0, C12:0, C20:0, C22:0, and C24:0. The ORs and CIs were as follows: for C4:0, an OR of 1.232 with a 95% CI of 0.891–1.703 and a *P*-value of 0.208; for C6:0, an OR of 1.262 with a 95% CI of 0.675–2.356 and a *P*-value of 0.466; for C8:0, an OR of 1.053 with a 95% CI of 0.724–1.532 and a *P*-value of 0.786; for C10:0, an OR of 1.222 with a 95% CI of 0.872–1.714 and a *P*-value of 0.244; for C12:0, an OR of 1.208 with a 95% CI of 0.991–1.473 and a *P*-value of 0.061; for C20:0, an OR of 1.34 with a 95% CI of 0.842–2.132 and a *P*-value of 0.217; for C22:0, an OR of 1.172 with a 95% CI of 0.759–1.809 and a *P*-value of 0.474; and for C24:0, an OR of 0.853 with a 95% CI of 0.544–1.336 and a *P*-value of 0.487. These subtypes are detailed in Supplementary File 3.

### Subgroup analysis

**Fig. 3:**
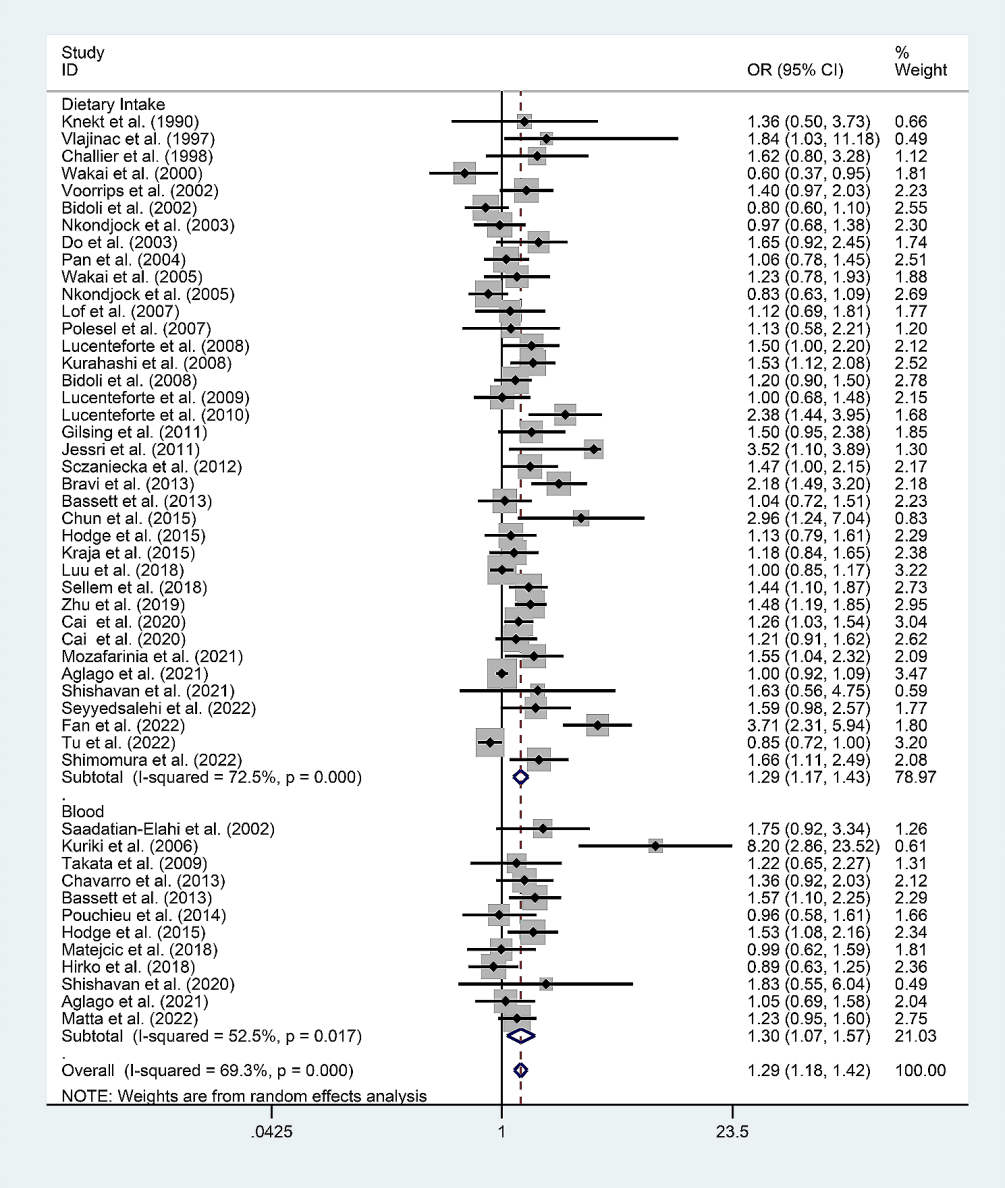
Subgroup analysis of the association between total SFA levels and the risk of cancer based on the source of SFAs (Dietary Intake/Blood)

Since high heterogeneity was revealed in the total SFA analysis (*I*^*2*^ = 69.3%), the subgroup analyses were carried out, stratified according to the origin of the total SFAs (dietary intake/blood) (Fig. 3). The analysis of the data on SFAs from dietary intake revealed that the risk of cancer due to high SFA intake (OR = 1.305; 95% CI: 1.172–1.453; *P* < 0.001) was 1.305 times that of low SFA intake. The analysis of the data on SFAs in the blood revealed that the risk of cancer associated with high levels of SFAs (OR = 1.27; 95% CI: 1.054–1.530; *P* = 0.012) was 1.27 times the risk of cancer associated with low levels of SFAs.

**Fig. 4:**
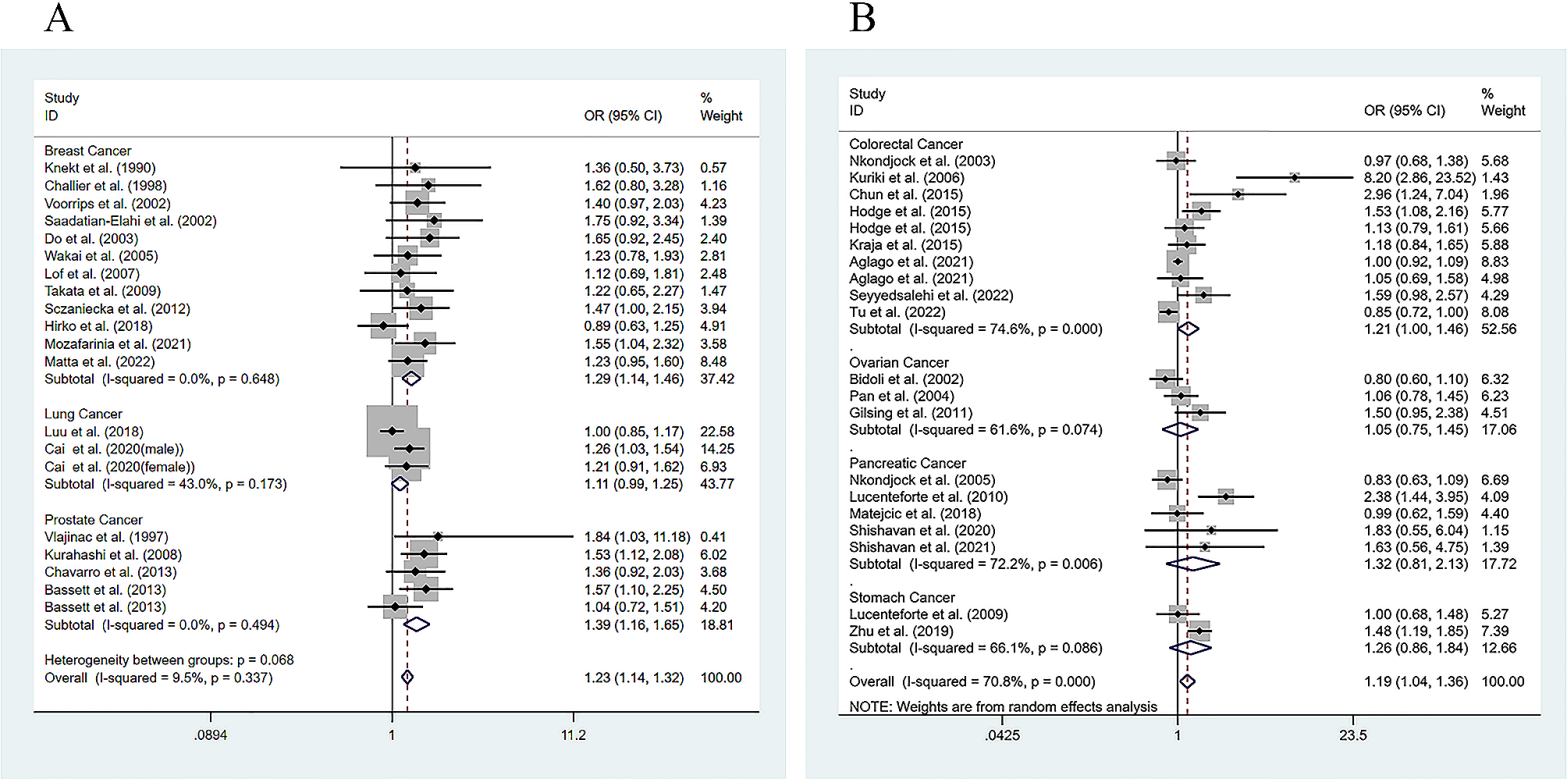
Meta-analysis for the association between total SFA levels and the cancer subtypes: (**A**) Breast cancer, lung cancer and prostate cancer; (**B**) colorectal cancer, ovarian cancer, pancreatic cancer, and stomach cancer

Subgroup analysis, differentiated by cancer type, demonstrated a significant positive association between increased levels of SFAs and the several cancers risk. Specifically, breast cancer exhibited an OR of 1.291 with a 95% CI from 1.140 to 1.462, and a *P*-value less than 0.001; prostate cancer showed an OR of 1.386 with a 95% CI from 1.163 to 1.651, and a *P*-value less than 0.001; and colorectal cancer had an OR of 1.211 with a 95% CI from 1.001 to 1.464, and a *P*-value of 0.049. In contrast, lung cancer (OR of 1.111; 95% CI: 0.991 to 1.246; *P*-value of 0.072), pancreatic cancer (OR of 0.830; 95% CI: 0.630 to 1.090; *P*-value of 0.266), ovarian cancer (OR of 1.046; 95% CI: 0.775 to 1.450; *P*-value of 0.788), and stomach cancer (OR of 1.259; 95% CI: 0.862 to 1.838; *P*-value of 0.233) did not show significant associations (Fig. 4A and B). Among the two studies on lung cancer, one study grouped the lung cancer patients according to gender. Therefore, the two groups of data with different genders were extracted separately and then analyzed (Fig. 4). No overlap between the two groups of people was noted, and the sample size had not been increased.

**Fig. 5:**
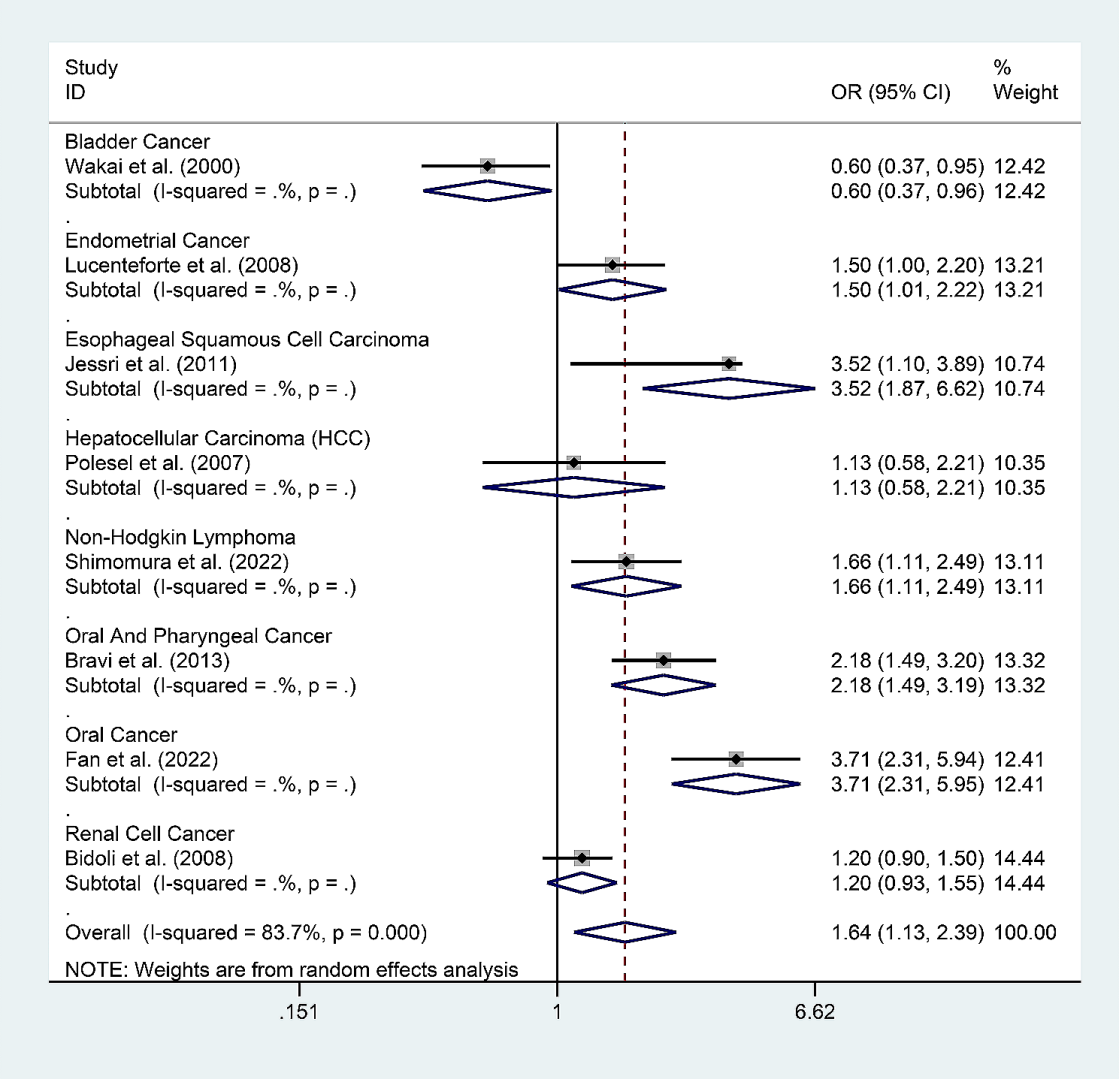
Meta-analysis for the association between the total SFA levels and cancer subtypes: bladder cancer, endometrial cancer, esophageal squamous cell carcinoma, hepatocellular carcinoma, non-hodgkin’s lymphoma, oral and nasopharyngeal carcinoma, oral cancer, and renal cell cancer

In addition, there was just one study each reporting bladder cancer, endometrial cancer, esophageal squamous cell carcinoma, hepatocellular carcinoma, non-Hodgkin’s lymphoma, oral and nasopharyngeal carcinoma, oral cancer, and renal cell carcinoma. The data in these studies suggested that high levels of SFAs exhibited a significant negative association with the risk of bladder cancer, a significant positive association with the risk of esophageal squamous cell carcinoma, non-Hodgkin’s lymphoma, oral and nasopharyngeal carcinoma, and oral cancer, and no correlation to the risk of endometrial cancer, hepatocellular carcinoma, or renal cell carcinoma **(**Fig. 5).

### Sensitivity and publication bias analysis

A comprehensive sensitivity analysis was conducted on the 55 articles, according to which all meta-analysis results were relatively stable. A publication bias analysis of these articles was conducted using Begg’s and Egger’s tests. Begg’s test *p*-value for total SFAs was 0.037, and Egger’s test *p*-value was 0.000, both indicating the presence of publication bias. Therefore, to address this bias, the trim-and-fill method was adopted, using which 13 articles were added. The adjusted results aligned with the original ones, indicating that the publication bias did not influence the final results (Supplementary File 4).

## Discussion

### Overall findings

This study represents a pioneering meta-analysis examining the correlation between SFA concentrations and cancer risk. This analysis indicates that elevated levels of total SFAs in the bloodstream are associated with an increased cancer risk. Similarly, excessive dietary intake of total SFAs appears to significantly amplify this risk. Notably, increased levels of total SFAs are linked to a heightened risk of specific cancers, including breast, prostate, and colorectal cancers. Furthermore, the particular SFA subtypes were observed, specifically C14:0, C16:0, and C18:0, are correlated with an elevated cancer risk. However, the existing literature on the relationship between different SFA subtypes and specific cancer types is limited.

### Fatty acids and cancer

Several studies have delineated the multiple roles of fatty acids in cancer cells, and the tumor microenvironment, and the number of such studies continues to increase. Fatty acids, in addition to being vital constituents of the membrane structure, serve as a source of energy, fueling the malignant proliferation of cancer cells, the metastasis of primary tumors, the establishment of secondary tumors, and impairment of the immune system. Fatty acids also function as signaling molecules, regulating the tumor microenvironment and facilitating signal transduction and epigenetic alterations, thereby creating conditions favorable to tumor progression. Concurrently, increased fatty acid synthesis, uptake, and oxidation provide the necessary energy and the associated molecules for the autonomous division, growth, and survival of tumor cells.

The human body primarily absorbs SFAs from the diet, and the incorporation of exogenous fatty acids renders the cancer cells metabolism more flexible [69]. Moreover, the exogenous fatty acids present in the tumor microenvironment reportedly promote cancer progression and survival [70]. The overconsumption of dietary SFAs is considered a factor contributing to obesity. Obesity, driven by a high-fat diet, reduces the number and the anti-tumor activity of CD8^+^ T cells within tumors, competes for lipid molecules, and accelerates tumor growth [71]. The present meta-analysis revealed that an excessive dietary intake of total SFAs increases the risk of cancer, although this does not imply that SFAs are invariably detrimental. A few SFAs are reported to inhibit certain types of cancers [72–75]. In addition to high levels of SFA in the diet, the other dietary components may also promote cancer. For instance, many studies have confirmed that red meat is associated with a variety of cancers. In addition to high levels of SFAs, red meat contains high levels of heme, which is difficult to be absorbed by the small intestine, and the excessive accumulation of heme in the colon induces colon damage and leads to colon cancer [76]. Moreover, Neu5Gc contained in red meat is not actively synthesized in the human body. When red meat is consumed, Neu5Gc promotes the production of antibodies, causes inflammation, and leads to the occurrence of colitis and colon cancer [77]. Recent studies have demonstrated that Neu5Gc, as a carcinogen, may stimulate the expression of the proto-oncogene HRAS and the PI3K-Akt pathway and accelerate cell cycle progression [78]. Benzopyrene and nitrite in processed meats and pickles also cause cancer. However, research exploring the link between dietary patterns rich in Saturated Fatty Acids (SFAs) and cancer risk remains limited. The ones that have been reported to date have investigated dietary patterns with high SFA content in relation to specific cancers, and all such studies have reported that this dietary pattern is positively associated with the risk of the concerned specific cancers [18, 19]. Consequently, an increasing number of patients, clinicians, and researchers are accepting the possibility of preventing the onset of cancer or improving the prognosis of cancer through the implementation of “anticancer” dietary interventions. Therefore, the effect of high dietary levels of SFAs interacting with other components of the diet on cancer warrants further investigation.

In addition, within the scope of this research, just one study reported the association of total SFAs with bladder cancer, endometrial cancer, esophageal squamous cell carcinoma, hepatocellular carcinoma, non-Hodgkin’s lymphoma, oral and nasopharyngeal carcinoma, oral cancer, and renal cell carcinoma [18, 34, 35, 39, 42, 43, 45, 54]. The conclusions drawn from the data in these studies could be used as a reference, although further research is warranted to verify the findings.

According to the saturation level of the constituting carbon chain, fatty acids are classified into SFAs and unsaturated fatty acids, and the latter are further divided into MUFAs and PUFAs based on the quantity of alkenes in the carbon skeleton.

The existing literature extensively investigates the association between unsaturated fatty acids and cancer. A large-scale study conducted with millions of participants reported no correlation between a high intake of MUFAs and cancer-related mortality, compared to the lowest MUFA intake [79]. A few studies reported specific relationships between certain MUFAs and cancer progression. For instance, the DGLA of MUFAs revealed that these could induce ferroptosis, thereby effectively leading to the death of cancer cells [80]. PUFAs undergo peroxidation under the toxicity of an acidic environment, thereby inducing ferroptosis in cancer cells and demonstrating anticancer efficacy [81].

Tumor cells take up exogenous fatty acids and activate de novo synthesis of fatty acids to meet the biosynthesis and energy requirements in the process of carcinogenesis and tumorigenesis [82]. After entering stearoyl-CoA desaturase 1 (SCD1), dietary SFAs are converted into unsaturated fatty acids under the action of stearoyl-CoA desaturase 1 (SCD1), which is used in the synthesis of phospholipids, triglycerides, and cholesterol esters. Studies show that SCD1 is highly expressed in a variety of human cancer tissues [83, 84]. The changes of lipid metabolism in cancer patients include the decrease of fat storage and the increase of fat utilization. In order to achieve rapid proliferation and meet their own energy needs, cancer cells promote the increase of de novo synthesis of fatty acids, resulting in dyslipidemia, increased oxidation of fatty acids, and the decrease of total fat. In cancer patients, the clearance rate of endogenous stored fat and exogenous intake fat increased in the state of fasting and feeding, and the liposolysis could not be inhibited after glucose intake, and the oxidation of fatty acids continued. Some studies have measured the fat clearance rate of patients with colon and rectal cancer, and found that the fat clearance rate of most patients increased, but remeasured 12 weeks after radical tumor resection, and found that the fat clearance rate of most patients was close to normal [85]. These indicate that cancer does not necessarily cause increased levels of SFA in the blood, and even in the later stages of cancer development, malnutrition and body fat loss become a major feature of cancer patients.

Previous studies on the relationship between SFAs and tumorigenesis are predominantly focused on the influence of palmitic acid on cancer, with an attempt to elucidate the mechanisms through which palmitic acid facilitates cancer progression [86]. In alignment with the conclusions drawn from previous research, this research also revealed a significant positive association between elevated palmitic acid levels and tumor incidence. In addition, it was revealed that SFAs such as C14:0 and C18:0 were positively associated with cancer risk. As the research in this direction progresses and deepens, the precise molecular mechanisms through which these fatty acids facilitate tumorigenesis are being gradually revealed, thereby laying the foundation for the identification of potential therapeutic targets to mitigate cancer metastasis. This research group is also exploring the correlation between changes in the plasma-free fatty acid profile post-obesity and tumor pathogenesis, identifying that both palmitic acid and oleic acid function as signaling molecules, accelerating the pathogenesis of endometrial and prostate cancers through the activation of GPR receptors specific to fatty acids [87, 88]. In addition, caprylic acid (C8:0) was revealed to enhance the bone metastasis of prostate cancer through changes in the adipocyte-osteoblast ratio in the bone marrow microenvironment [89]. These findings offer valuable insights into the relevant mechanisms, which remain unknown to date, through which specific fatty acids promote the pathogenesis of specific tumors.

This meta-analysis primarily examines the link between dietary fatty acid intake and tumor incidence. Current literature underscores obesity as a significant risk factor in the onset and progression of various tumors. This is primarily attributed to the enhanced lipid metabolism following obesity, which induces changes in the plasma free fatty acid profile, thereby causing lipotoxicity due to the abnormal elevation in the levels of certain fatty acids. Notably, meta-analyses exploring the correlation among obesity, elevated levels of plasma fatty acids, and tumor pathogenesis are scarce and, therefore, could represent a critical direction for future research.

Certain findings of the present study were different from those reported in the literature. While a few studies have reported the role of butyric acid in combating colon cancer [90–92], the present study revealed no association between elevated butyric acid levels and the risk of cancer. According to certain previous research, propionic acid could inhibit breast and colorectal cancers [73, 74] and valeric acid could inhibit prostate and breast cancers [72, 75]. However, in the present meta-analysis, propionic and valeric acids were not evaluated for their association with cancer due to the unavailability of sufficient data in the literature, which if used, could have yielded inconclusive results.

According to the pooled data from the current research, total SFAs exhibited no correlation with the pancreatic cancer risk (OR of 0.830; 95% CI: 0.630–1.090; *P-*value of 0.266), which was inconsistent with certain previous studies that have reported inhibitory effects of palmitic acid ester and stearic acid ester on pancreatic cancer cell proliferation [93] and propose that SFAs promote the growth of cancer cells [94].

These disparities between the findings of the present study and those reported in the literature could be due to marked variations in the amount and quality of the SFAs intake by different subjects across different studies. In large-scale studies, such as the European cohort study, statistical data often rely on the subjects’ self-reported SFA intake, which could be significantly different from the SFA data acquired through direct measurements conducted for the individuals. Furthermore, the impact of various SFAs may differ across the different malignancies.

### Limitations of the study and implications for future research

The association between SFAs and the risk of cancer remains debatable to date in the scientific community. Specifically in regard to the underlying mechanisms, considering the various SFA subtypes and kinds of cancer, the structure of SFAs could influence their activity, causing them to possibly exert unique effects during different stages in various cancers. This also demonstrates, to a certain extent, the credibility of the conclusions drawn in the present study.

Nonetheless, as with all research, the present also has certain limitations. The large heterogeneity in the data was mainly due to two key factors: (1) The included data that were based on the dietary intake were typically determined through surveys gauging the intake of SFAs by individuals. In the data collection process, while certain surveys involved interviews conducted by trained personnel, others involved data collected simply through questionnaires completed by participants themselves, and the latter could introduce recall bias and measurement inaccuracies, resulting in detection bias. (2) In cohort studies, variations among the study subjects in terms of ethnicity, gender, geographic location, and the duration of follow-up also contributed significantly to data heterogeneity. In similar studies to be conducted in the future, a more objective method should be adopted to overcome these limitations regarding the assessment of SFA intake through the use of biomarkers for the measurement of short-term concentrations of SFAs in the blood [95].

This study stands as the first comprehensive meta-analysis exploring the correlation with SFA content and cancer risk, thereby marking a significant contribution to the growing field of dietary modifications for cancer prevention and management. The findings offer potential strategies for cancer prevention and improving outcomes in cancer patients. The mechanistic interplay between SFAs and cancer warrants further investigation, in which it would be crucial to consider the interactions among SFAs, the tumor microenvironment, obesity, and other nutrients to gather robust evidence substantiating the link between SFAs and cancer. Such endeavors would provide novel and further efficient strategies for the prevention and adjuvant-based treatment of cancer.

## Conclusion

The present study is a systematic review that demonstrates the association of high levels of SFAs with an elevated risk of cancer, thereby indicating that high SFA levels could have potentially detrimental effects on the health of cancer patients. Despite the limitations of the present study due to the heterogeneity of data sources, its findings did offer pertinent insights. Future investigations into the link between SFAs and cancer risk should incorporate more sophisticated research methodologies, including the use of biomarkers for SFA intake assessment and adjustments for major confounders. Additionally, the distinct impacts of various SFA subtypes warrant targeted prevention strategies. Accordingly, future research could focus on which SFA subtypes could exhibit higher carcinogenicity and which populations could be at a higher risk of cancer due to increased sensitivity to SFAs. In summary, the findings of the present study would serve as evidence on the role of dietary SFA intake in cancer and be useful when providing dietary recommendations for cancer prevention and management. Accordingly, it is suggested that clinical trials conducted using a combination of diet-related interventions and immunotherapy to improve outcomes in cancer patients might be worth considering.

### Electronic supplementary material

Below is the link to the electronic supplementary material.

**Supplementary Material 1: Supplementary Table 1.** Results of all the included studies.

**Supplementary Material 2: Supplementary Table 2.** The use of the Newcastle-Ottawa Scale for assessing the quality of the included articles.

**Supplementary Material 3: Supplementary File 1.** PRISMA_2020_checklist.

**Supplementary Material 4: Supplementary File 2.** Search history.

**Supplementary Material 5: Supplementary File 3.** Meta-analysis for the SFA subtypes and cancer.

**Supplementary Material 6: Supplementary File 4.** Additional analyses: Begg’s test; Egger’s test; Sensitivity analysis; Publication bias evaluation.

## Data Availability

All data generated or analyzed during this study are included in this published article (and its Supplementary Information files).

## Abbreviations

SFAs: Saturated Fatty Acids
WCRF/AICR: World Cancer Research Fund/American Institute for Cancer Research
PRISMA: Preferred Reporting Items for Systematic reviews and Meta-Analysis
PICOS: Population, Intervention, Comparison, Outcome, and Study design
OR: Odds Ratio
RR: Relative Risk
HR: Hazard Ratio
CI: 95% Confidence Interval
NOS: Newcastle-Ottawa Scale
MUFAs: Monounsaturated Fatty Acids
PUFAs: Polyunsaturated Fatty Acids
DGLA: Dihomo-Gamma-Linolenic Acid
Neu5Gc: N-Glycolylneuraminic acid
HRAS: HRAS proto-oncogene
PI3K: Phosphatidyl-Inositide-3-kinases
Akt (PKB): protein kinase B
SCD1: Stearoyl-CoA Desaturase 1
