# Supplementary material for: Association of saturated fatty acids with cancer risk: a systematic review and meta-analysis
Source: Lipids Health Dis. 2024 Jan 30;23:32. doi: 10.1186/s12944-024-02025-z (PMC10826095; doi:10.1186/s12944-024-02025-z)
Supplement: Supplementary file 1 — Supplementary Material 1: Supplementary Table 1. Results of all the included studies. [file 12944_2024_2025_MOESM1_ESM.docx]

Supplementary Table 1 Results of all the included studies

| Author Year Country | SFAs test source | OR/RR/HR(95% CI)，*p*-value | Meaning of OR/RR/HR | Cancer | Adjusted factors | Notes |
| --- | --- | --- | --- | --- | --- | --- |
|  |  |  |  |  |  |  |
| Wu et al. 2023 China | Blood | (1)OR 1.94 (1.38,2.74) Trend:<0.001 (2)OR 2.35 (1.64,3.35) Trend:<0.001  (3)OR 2.30 (1.60,3.30) Trend:<0.001 (4)OR 0.72(0.52,1.00) Trend:<0.001 (5)OR 0.72(0.52,1.00) Trend:<0.001 | (1)C12:0: Q4/Q1  (2)C17:0: Q4/Q1  (3)C20:0 :Q4/Q1  (4)C22:0: Q2/Q1  (5)C24:0: Q2/Q1 | Colorectal | Sex, age, residence, type of job, education, marital status, income, working physical activities, household and leisure-time physical activities, smoking, passive smoking, drinking, family history of cancer in first-degree relatives, BMI, total energy intake, dietary red and processed meat intake, dietary calcium intake, dietary fibre intake, serum MUFAs, and serum PUFAs. |  |
| Nkondjock et al. 2003 Canada | Dietary Intake | (1)OR 0.97 (0.68,1.38) Trend:0.525 (2)OR 0.84 (0.59,1.20) Trend:0.038 (3)OR 0.74 (0.52,1.05) Trend:0.059 (4)OR 0.83(0.58,1.18) Trend:0.166 (5)OR 0.96 (0.68,1.35) Trend:0.099 (6)OR 0.89 (0.63,1.26) Trend:0.104 (7)OR 1.03 (0.72,1.48) Trend:0.746 (8)OR 1.04 (0.72,1.48) Trend:0.777 | (1)Total SFA: Q4/Q1  (2)C4:0: Q2/Q1  (3)C8:0: Q4/Q1  (4)C10:0: Q3/Q1  (5)C12:0: Q2/Q1 (6)C14:0: Q2/Q1 (7)C16:0: Q4/Q1 (8)C18:0: Q4/Q1 | Colorectal | Age, marital status, history of colorectal cancer in first-degree relatives, body mass index one year prior to diagnosis, and physical activity |  |
| Mozafarinia et al. 2021 Iran | Dietary Intake | OR 1.55 (1.04,2.32) Trend:0.11 | Total SFA: Q3/Q1 | Breast | Age and energy,cigar smoking, marital status, alcohol consumption, physical activity, education, family history of breast cancer, parity, and BMI |  |
| Seyyedsalehi et al. 2022 Iran | Dietary Intake | (1)OR 1.59 (0.98,2.57) Trend:0.081 (2)OR 1.19 (0.76,1.87) Trend:0.28 (3)OR 0.99 (0.34,2.89)  Trend:0.234 (4)OR 1.01 (0.40,2.52) Trend: 0.695 (5)OR 0.76 (0.47,1.24) Trend: 0.034 (6)OR 1.28 (0.78,2.09)  Trend: 0.695 | (1)Total SFA: Q4/Q1  (2)C14:0: Q2/Q1  (3)C15:0: Q3/Q1  (4)C16:0: Q4/Q1  (5)C17:0: Q2/Q1 (6)C18:0: Q2/Q1 | Colorectal | Province, age, SES, gender, BMI, tobacco use, opium use, aspirin use, physical activity, processed meat, fiber intake, calcium, and energy intake |  |
| Fan et al. 2022 China | Dietary Intake | OR 3.71 (2.31, 5.94) Trend:<0.001 | Total SFA: Q3/Q1 | Oral | Demographic characteristics and tobacco smoking, drinking, oral hygiene score |  |
| Tu et al. 2022 China | Dietary Intake | OR 0.85 (0.72.1.00) Trend:<0.001 | Total SFA: Q2/Q1 | Colorectal | Age (years), marital status, residence, ducation, occupation, income, occupational activity |  |
| Cai et al. 2020 Japan | Dietary Intake | (1)HR 1.26 (1.03,1.54) Trend:0.035 (2)HR 1.21 (0.91,1.62) Trend:0.270 | (1)Total SFA: Q4/Q1(men) (2)Total SFA: Q3/Q1(women) | Lung | Age, area, total energy intake, smoking status (never, past, 1-19, 20-29, 30-39, 40-49, 50-59, over 60 pack-year for current smokers in men; never, past, 1-19, over 20 pack-year for current smokers in women), alcohol intake (nondrinkers, 1-3 times/month, 1-150, 150-300, 300-450, over 450 g ethanol/week in men; nondrinker, 1-150, over 150 g ethanol/week in women), physical activity (almost never, 1-2 times/month, ≥1 time/week),body mass index (<23, 23-25, 25-27, ≥27 kg/m2). | Male |
|  |  |  |  |  | Age, area, total energy intake, smoking status, alcohol intake, physical activity, body mass index, vegetables (quartile), fruit (quartile), for meat groups, further adjusted for unprocessed red meat, processed red meat, poultry, fish mutually. | Female |
| Shimomura et al. 2022 Japan | Dietary Intake | HR 1.66 (1.11, 2.49) Trend:0.074 | Total SFA: Q4/Q1 | Non-Hodgkin Lymphoma | Age , sex, study area, BMI, history of smoking, alcohol consumption frequency, and physical activity by metabolic equivalents per day. |  |
| Takata et al. 2009 USA | Blood | (1)OR 1.22 (0.65,2.27) Trend:0.42 (2)OR 0.96 (0.52,1.79) Trend:0.87 (3)OR 1.24 (0.64,2.40) Trend:0.41 (4)OR 1.35 (0.73,2.49) Trend:0.74 (5)OR 1.47 (0.79,2.75) Trend:0.10 (6)OR 1.65 (0.87,3.13) Trend:0.07 | (1)Total SFA: Q4/Q1  (2)C14:0: Q4/Q1  (3)C16:0: Q4/Q1 (4)C18:0: Q3/Q1 (5)C22:0: Q4/Q1 (6)C24:0: Q3/Q1 | Breast | Age, study center, year of the enrollment, smoking status at baseline and blood draw (current vs. former), BMI [normal (\25 kg/m2), overweight (25.0–30 kg/m2), or obese (C30 kg/m2)], intervention arm (supplementation vs. placebo), and alcohol use at the time of blood draw [none, 0–1 drink/day (B15 g of ethanol), or C1 drinks/day]. |  |
| Chun et al. 2015 Korea | Dietary Intake | OR 2.96 (1.24, 7.04) Trend:0.016 | Total SFA: T3/T1 | Colorectal | Energy intake (in kcal/d; < 1,620, 1,620-2,250, or ≥ 2,250), sex (male, female), age (in year; 20-29, 30-39, 40-49, 50-59, 60-69, or 70-79), household income (in 10,000won; < 100, 100-200, 200-400, or ≥ 400), education (≤ elementary school, middle school, high school, ≥ university), smoking (none, ex/current), alcohol drinking frequency (in times/month; none, < 1, 1-3, 4-11, or ≥ 12), exercise frequency (in times/week; none, 1-3,or ≥ 4), BMI (in kg/m2;<18.5-22.9, 23-24.9, 25-29.9, or ≥ 30),dietary fiber (in g/d; < 27.5, 27.5-45, or > 45) |  |
| Jackson et al. 2012 Jamaica | Blood | (1)OR 2.58 (0.65, 6.26) Trend:0.177 (2)OR 0.53 (0.19, 1.54)  Trend:0.061 (3)OR 1.48 (0.89, 2.44) Trend:0.115 | (1)C14:0: Q3/Q1 (2)C16:0: Q3/Q1 (3)C18:0: Q3/Q1 | Prostate | Age, family history of prostate cancer, education, smoking and body mass index |  |
| Chavarro et al. 2013 USA | Blood | (1)RR 1.36 (0.92,2.03) Trend:0.75 (2)RR 1.70 (1.10,2.64) Trend:0.40 (3)RR 1.28(0.83,1.96) Trend:0.25 (4)RR 1.02 (0.69,1.50) Trend:0.10 | (1)Total SFA: Q2/Q1  (2)C14:0: Q4/Q1  (3)C16:0: Q4/Q1  (4)C18:0: Q3/Q1 | Prostate | Age, smoking status at baseline, and length of follow-up |  |
| Nkondjock et al. 2003 Canada | Dietary Intake | (1)OR 1.41 (0.94,2.12) Trend:0.680 (2)OR 1.07 (0.72,1.60) Trend:0.941 (3)OR 1.23 (0.82,1.85) Trend:0.561 (4)OR 1.16 (0.78,1.73) Trend:0.841 (5)OR 1.06 (0.71,1.59) Trend:0.682 (6)OR 1.20 (0.81,1.80) Trend:0.738 (7)OR 1.30 (0.87,1.94) Trend:0.912 | (1)C4:0: Q2/Q1  (2)C8:0: Q2/Q1  (3)C10:0 :Q2/Q1  (4)C12:0: Q3/Q1  (5)C14:0: Q2/Q1 (6)C16:0: Q3/Q1 (7)C18:0: Q2/Q1 | Breast | Age at first full-term pregnancy, history of BC in first-degree relatives, history of benign breast disease, number of full-term pregnancies, smoking, marital status, and total energy intake. |  |
| Pan et al. 2004 Canada | Dietary Intake | OR 1.06 (0.78, 1.45) Trend:0.45 | Total SFA: Q4/Q1 | Ovarian | 10-year age group, province of residence, education, alcohol consumption, cigarette pack-years, BMI, total caloric intake, recreational physical activity, number of live births, menstruation years, and menopause status |  |
| Shishavan et al. 2020 Iran | Blood | (1)OR 1.83 (0.55,6.04) Trend:0.09 (2)OR 7.02 (1.33,36.87) Trend:0.017 (3)OR 1.00 (0.33,3.03) Trend:0.104 (4)OR 0.10 (0.01,0.53) Trend:0.067 | (1)Total SFA: Q2/Q1  (2)C14:0: Q4/Q1  (3)C16:0: Q2/Q1  (4)C18:0: Q2/Q1 | Pancreatic | Energy and total fat intake, BMI, age, gender, marital status, residence, smoking, opuim consumption, diabetes, physical activity, family cancer, ethnicity, wealth score and education. |  |
| Matta et al. 2022 USA | Blood | (1)OR 1.23 (0.95,1.60) Trend:0.06 (2)OR 1.51 (1.17,1.93) Trend:<0.001 (3)OR 1.00 (0.79,1.28) Trend:0.28 (4)OR 1.42 (1.08,1.87) Trend:0.021 (5)OR 0.79 (0.62,1.01) Trend:0.05 (6)OR 0.86 (0.67-1.10) Trend:0.12 | (1)Total SFA: Q4/Q1  (2)C14:0: Q4/Q1  (3)C15:0: Q4/Q1 (4)C16:0: Q4/Q1 (5)C17:0: Q4/Q1 (6)C18:0: Q3/Q1 | Breast | Smoking status (never, former, current), alcohol use (no, yes, missing), hormone replacement therapy group (never user, current oestrogen only, former oestrogen only, current oestrogen + progesterone, former oestrogen + progesterone, other/unknown), waist circumference (inches; continuous), weight change from age 18 to blood draw (weight loss: >5 lbs, stable weight: ±5 lbs, weight gain: >5-20 lbs, weight gain: >20-40 lbs, weight gain: >40-60 lbs, weight gain: >60 lbs, missing), BMI at blood draw (in kg/m2) (<18.5, 18.5 to ≤25, 25 to <30, 30+, missing), and 3 diet component PCA variables. |  |
| Matejcic et al. 2018 Denmark、France、Greece、Germany、Italy、Netherlands、Norway、Spain、Sweden、UK | Blood | (1)OR 0.99 (0.62,1.59) Trend:0.977 (2)OR 0.88 (0.56,1.37) Trend:0.058 (3)OR 1.05 (0.69,1.59) Trend:0.787 (4)OR 0.63 (0.41,0.98) Trend:0.036 (5)OR 0.92 (0.61,1.38) Trend:0.686 | (1)Total SFA: T3/T1  (2)C15:0: T2/T1  (3)C16:0: T3/T1  (4)C17:0: T3/T1  (5)C18:0: T3/T1 | Pancreatic | Body mass index, height, history of diabetes mellitus, smoking status, alcohol intake, education, and physical activity |  |
| Gilsing et al. 2011 Netherlands | Dietary Intake | RR 1.50 (0.95, 2.38) Trend:0.12 | Total SFA: Q5/Q1 | Ovarian | Age (y) and total energy intake (kcal) |  |
| Kurahashi et al. 2008 Japan | Dietary Intake | (1)RR 1.53 (1.12,2.08) Trend:0.01 (2)RR 1.82 (1.34,2.47) Trend:<0.01 (3)RR 1.65 (1.20,2.28) Trend:<0.01 (4)RR 1.46 (1.05,2.02) Trend:0.03 | (1)Total SFA: Q4/Q1  (2)C14:0: Q4/Q1  (3)C16:0: Q4/Q1  (4)C18:0: Q4/Q1 | Prostate | Age-area |  |
| Vlajinac et al. 1997 Serbia | Dietary Intake | OR 1.84 (1.03,11.18) Trend:NS | Total SFA: T3/T1 | Prostate | Nutrients which are significant when unadjusted or adjusted for energy |  |
| Hodge et al. 2015 Italian and Greek | Blood | (1)HR 1.53 (1.08, 2.16) Trend:0.005 (2)HR 1.34 (0.96, 1.87) Trend:0.64 (3)HR 0.96 (0.71, 1.30) Trend:<0.001 (4)HR 1.58 (1.10, 2.27) Trend:0.007 (5)HR 1.00 (0.70, 1.42) Trend:0.88 | (1)Total SFA: Q5/Q1  (2)C14:0: Q2/Q1  (3)C15:0: Q2/Q1  (4)C16:0: Q4/Q1 (5)C18:0: Q2/Q1 | Colorectal | Education, alcohol intake, smoking status, physical activity, total energy intake and stratified by: sex, ethnicity (Southern-European migrant vs. not) and family history of cancer. |  |
|  | Dietary Intake | (1)HR 1.13 (0.79, 1.61) Trend:0.81 (2)HR 1.31 (0.93, 1.85) Trend:0.45 (3)HR 1.31 (0.93, 1.84) Trend:0.28 (4)HR 1.08 (0.76, 1.52) Trend:0.65 (5)HR 1.15 (0.81, 1.63) Trend:0.98 | (1)Total SFA: Q3/Q1  (2)C14:0: Q3/Q1  (3)C15:0: Q2/Q1  (4)C16:0: Q5/Q1 (5)C18:0: Q2/Q1 |  |  |  |
| Lof et al. 2007 Sweden | Dietary Intake | HR 1.12 (0.69,1.81) Trend:0.43 | Total SFA: Q5/Q1 | Breast | Education, parity, age at menarche, use of oral contraceptives, age at first birth by parity, first-degree relative with breast cancer, non-alcohol total energy intake, total fat intake, BMI and alcohol intake |  |
| Knekt et al. 1990 Finland | Dietary Intake | RR 1.36 (0.50, 3.73) Trend:0.31 | Total SFA: Q3/Q1 | Breast | Age and energy |  |
| Thiébaut et al. 2009 USA | Dietary Intake | (1)HR 1.33 (1.11,1.58) Trend:<0.001 (2)HR 1.31 (1.10,1.56) Trend:0.008 | (1)C16:0: Q5/Q1  (2)C18:0: Q5/Q1 | Pancreatic | Total energy intake (continuous), smoking history (never smoked; quit ≥ 10 years ago; quit 5 – 9 years ago; quit 1 – 4 years ago; quit <1 year ago or current smoker with ≤ 20 cigarettes per day; quit <1 year ago or current smoker with >20 cigarettes per day;or missing), body mass index (<18.5, 18.5 to <25, 25 to <30, 30 to <35, ≥ 35 kg/m 2,or missing),and self-reported history of diabetes (yes, no). |  |
| Kraja et al. 2015 Netherlands | Dietary Intake | HR 1.18 (0.84, 1.65) Trend:0.52 | Total SFA: T2/T1 | Colorectal | Age, gender, energy-adjusted DF intake, and Dutch Healthy Diet index (excluding PUFA, fish, SFA, and DF components). |  |
| Aglago et al. 2021 Denmark、France、Greece、Germany、Italy、Netherlands、Norway、Spain、Sweden、UK | Dietary Intake | (1)HR 1.00(0.92,1.09) Trend:0.006 (2)HR 0.95 (0.88,1.04) Trend:0.002 (3)HR 1.05 (0.95,1.17) Trend:0.479 (4)HR 0.99 (0.91,1.07) Trend:0.017 (5)HR 1.13 (1.04,1.23) Trend:0.515 (6)HR 1.00 (0.92,1.09) Trend:0.095 | (1)Total SFA: Q2/Q1  (2)C14:0: Q3/Q1  (3)C15:0: Q5/Q1  (4)C16:0: Q2/Q1  (5)C17:0: Q3/Q1 (6)C18:0: Q2/Q1 | Colorectal | BMI (continuous), height (continuous), physical activity (inactive, moderately inactive, moderately active, active), smoking (never, 1-15 cigarettes/day, 16-25 cigarettes/day, over 26 cigarettes/day, former smokers who quit <10 years, former smokers who quit 11-20 years, former smokers who quit >20 years, current pipe-cigar and occasional smokers), education (none, primary, technical and professional, secondary, higher education), and dietary intakes of energy (continuous), red and processed meats (continuous), fibre (continuous), alcohol (continuous), and calcium (continuous) and stratified by age, sex, and centre. |  |
|  | Blood | (1)HR 1.05 (0.69,1.58) Trend:0.952 (2)HR 0.69 (0.45,1.05) Trend:0.005 (3)HR 0.79 (0.53,1.18) Trend:0.105 (4)HR 1.02 (0.68,1.53) Trend:0.396 (5)HR 0.91 (0.58,1.44) Trend:0.539 (6)HR 1.69 (1.07,2.65) Trend:0.087 | (1)Total SFA: Q2/Q1  (2)C14:0: Q2/Q1  (3)C15:0: Q2/Q1  (4)C16:0: Q2/Q1  (5)C17:0: Q3/Q1 (6)C18:0: Q2/Q1 |  | BMI (continuous), height (continuous), physical activity (inactive, moderately inactive, moderately active, active), smoking (never, 1-15 cigarettes/day, 16-25 cigarettes/day, over 26 cigarettes/day, former smokers who quit <10 years, former smokers who quit 11-20 years, former smokers who quit >20 years, current pipe-cigar and occasional smokers), education (none, primary, technical and professional, secondary, higher education), and dietary intakes of energy (continuous), red and processed meats (continuous), fibre (continuous), alcohol (continuous), and calcium (continuous). |  |
| Shishavan et al. 2021 Iran | Dietary Intake | (1)HR 1.63(0.56,4.75) Trend:0.01 (2)HR 1.001(0.51,1.92) Trend:0.35 (3)HR 0.81(0.43,1.53) Trend:0.04 (4)HR 0.70(0.37,1.32) Trend:0.01 (5)HR 1.09(0.56,2.13) Trend:0.55 (6)HR 1.13(0.58,2.18) Trend:0.37 (7)HR 1.17(0.57,2.43) Trend:0.78 (8)HR 0.72(0.32,1.63) Trend:0.30 (9)HR 2.57(1.03,6.41) Trend:0.01 (10)HR 1.25(0.53,2.92) Trend:0.33 (11)HR 1.80(0.76,4.28) Trend:0.01 (12)HR 0.42(0.22,0.81) Trend:<0.001 | (1)Total SFA: Q4/Q1 (2)C4:0: Q2/Q1 (3)C6:0: Q3/Q1 (4)C8:0: Q2/Q1 (5)C10:0: Q3/Q1 (6)C12:0: Q3/Q1 (7)C14:0: Q3/Q1 (8)C16:0: Q3/Q1 (9)C18:0: Q4/Q1 (10)C20:0: Q4/Q1 (11)C22:0: Q4/Q1 (12)C24:0: Q4/Q1 | Pancreatic | Energy and total fat intake, BMI, age, gender, marital status, residence, smoking, diabetes, physical activity, opium consumption, family history of cancer, ethnicity, wealth score and education |  |
| Zhu et al. 2019 China | Dietary Intake | OR 1.48 (1.19, 1.85) Trend:0.005 | Total SFA:Q3/Q1 | Stomach | Study area, age (continuous), gender (male/female), education level (illiterate, primary school, middle school, high school or above), income 10 years ago (<1000, 1000 to <1500, 1500 to <2500, ≥2500), smoking (continuous, pack-years), alcohol consumption (continuous, g ethanol/day), family history of stomach cancer (yes/no), H. pylori infection (positive/negative), BMI (<18.5, 18.5 to <24, 24 to <28, ≥28), exercise 10 years ago (yes/no), dietary sodium intake (quartile levels of raw for aOR and energy-adjusted for rOR) and total energy intake (continuous, kcal/day); |  |
| Sczaniecka et al. 2012 USA | Dietary Intake | (1)HR 1.47 (1.00, 2.15) Trend:0.09 (2))HR 1.68 (1.13, 2.50) Trend:0.02 (3))HR 1.65 (1.12, 2.43) Trend:0.03 | (1)Total SFA: Q4/Q1  (2)C16:0: Q4/Q1 (3)C18:0: Q4/Q1 | Breast | Age, race, education, height, body mass index, age at menarche, age at first birth, age at menopause, history of hysterectomy,years of combined hormone therapy, years of estrogen hormone therapy, family history of breast cancer, mammography, history of benign breast biopsy, regular use of nonsteroidal antiinflammatory drugs, exercise, alcohol consumption, vegetable intake, fruit intake, and total energy |  |
| Wakai et al. 2005 Japan | Dietary Intake | RR 1.23 (0.78, 1.93) Trend:0.21 | Total SFA:Q2/Q1 | Breast | Age |  |
| Shannon et al. 2007 China | Blood | (1)OR 2.20 (1.22, 3.96) Trend:0.0003 (2)OR 1.57 (0.79, 3.13) Trend:0.83 | (1)C16:0: Q2/Q1  (2)C18:0: Q2/Q1 | Breast | Age, duration of breastfeeding, age at first birth, time since last induced abortion, and duration of intrauterine device use. | 18:00 |
|  |  |  |  |  | Age | 16:00 |
| Hirko et al. 2018 USA | Blood | (1)RR 0.89 (0.63–1.25)  Trend:0.47 (2)RR 1.12 (0.77–1.63)  Trend:0.48 (3)RR 0.91 (0.63–1.31)  Trend:0.80 (4)RR 1.21 (0.87–1.69)  Trend:0.95 (5)RR 1.02 (0.71–1.46)  Trend:0.19 (6)RR 1.23 (0.87–1.74)  Trend:0.75 (7)RR 1.21 (0.85–1.71) Trend:0.94 (8)RR 1.18 (0.85–1.64)  Trend:0.89 (9)RR 1.34 (0.93–1.93)  Trend:0.81 (10)RR 1.05 (0.71–1.55)  Trend:0.34 | (1)Total SFA: Q3/Q1  (2)C12:0: Q3/Q1  (3)C14:0: Q5/Q1  (4)C15:0: Q2/Q1  (5)C16:0: Q3/Q1  (6)C17:0: Q2/Q1 (7)C18:0: Q2/Q1 (8)C20:0: Q2/Q1 (9)C22:0: Q3/Q1 (10)C24:0: Q3/Q1 | Breast | Age at menarche (<12, 13–14+years), age at first birth/parity (nulliparous, 1–2 children <25 years, 3+children <25 years, 1–2 children ≥25 years, and 3+ children ≥25 years), lactation (yes/no), family history of breast cancer (yes/no), history of benign breast disease (yes/ no), alcohol consumption(</≥5 grams/day), BMI at age 18 (<21, 21–23 and ≥23 kg/m2), weight change between age 18 and blood collection(continuous), physical activity (<3, 3 to <9, 9 to <18, 18 to <27 and 27+MET-hr/week). |  |
| Crowe et al. 2008 Denmark,Germany, Greece, Italy, Netherlands, Spain, Sweden, and UK | Blood | (1)RR 1.12 (0.79, 1.59) Trend: 0.322 (2)RR 1.49 (1.05, 2.12) Trend: 0.032 | (1)C14:0: Q5/Q1  (2)C16:0: Q3/Q1 | Prostate | BMI, smoking, alcohol intake, education, marital status, and physical activity |  |
| Wakai et al. 2000 Japan | Dietary Intake | OR 0.60 (0.37,0.95) Trend:0.032 | Total SFA: Q4/Q1 | Bladder | Smoking and occupational history as a cook |  |
| Bravi et al. 2013 Italy and Switzerland | Dietary Intake | OR 2.18 (1.49,3.20) Trend:0.0003 | Total SFA: Q4/Q1 | Oral and Pharyngeal | Age, sex, centre, education, year of interview, body mass index, tobacco smoking and alcohol drinking. |  |
| Challier et al. 1998 French | Dietary Intake | OR 1.62(0.80,3.28) Trend:0.06 | Total SFA: Q5/Q1 | Breast | Total calory intake, parity, weight, and corporeal surface |  |
| Voorrips et al. 2002 Netherlands | Dietary Intake | (1)RR 1.40 (0.97, 2.03) Trend:0.11 (2)RR 1.15 (0.87, 1.53) Trend:0.63 (3)RR 1.18 (0.90, 1.56) Trend:0.66 | (1)Total SFA: Q5/Q1  (2)C16:0: Q3/Q1  (3)C18:0: Q3/Q1 | Breast | Age, history of benign breast disease, maternal breast cancer, breast cancer in one or more sisters, age at menarche, age at menopause, oral contraceptive use, parity, age at first childbirth, Quetelet index, education, alcohol use, current cigarette smoking, total energy intake, and total energyadjusted fat intake. | Total SFA |
|  |  |  |  |  | Age | 16:0、18:0 |
| Do et al. 2003 Korean | Dietary Intake | OR 1.65(0.92, 2.45) Trend:0.0458 | Total SFA: Q3/Q1 | Breast | Age at menarche, total menstrual periods (years), pregnancy, total number of full term delivery, total periods of breast feeding (total months), family history of breast cancer and current BMI (body mass index). |  |
| Gong et al. 2010 USA | Dietary Intake | (1)OR 2.6 (1.9,3.5) Trend:<0.0001 (2)OR 2.3 (1.7,3.2) Trend:<0.0001 (3)OR 2.1 (1.6,2.9) Trend:<0.0001 (4)OR 2.2 (1.6,2.9) Trend:<0.0001 (5)ORR 1.7 (1.3,2.2) Trend:0.0004 (6)OR 2.2 (1.6,2.9) Trend:<0.0001 (7)OR 2.1 (1.6,2.7) Trend:<0.0001 (8)OR 2.3 (1.7,3.1) Trend:<0.0001 | (1)C4:0: Q4/Q1  (2)C6:0: Q4/Q1  (3)C8:0: Q4/Q1  (4)C10:0: Q4/Q1  (5)C12:0: Q4/Q1 (6)C14:0: Q4/Q1 (7)C16:0: Q4/Q1 (8)C18:0: Q4/Q1 | Pancreatic | Age in 5-year groups, sex and total energy intake (quartiles). | 12:0、14:0、16:0、18:0 |
|  |  |  |  |  | Race, education, body mass index, history of diabetes, smoking, physical activity and alcohol consumption. | 4:0、6:0、8:0、10:0 |
| Lucenteforte et al. 2008 Italy | Dietary Intake | OR 1.50 (1.00,2.20) Trend:0.08 | Total SFA: Q4/Q1 | Endometrial | Year of interview, education, physical activity, body mass index, history of diabetes, age at menarche, age at menopause, parity, oral contraceptives use, hormone replacement therapy use, total energy intake |  |
| Lucenteforte et al. 2009 Italy | Dietary Intake | OR 1.00 (0.68,1.48) Trend:0.79 | Total SFA: Q2/Q1 | Stomach | Year of interview, education, physical activity, body mass index, tobacco smoking, family history of stomach cancer and total energy intake (when appropriate) |  |
| Lucenteforte et al. 2010 Italy | Dietary Intake | OR 2.38 (1.44,3.95)  Trend:0.659 | Total SFA: Q3/Q1 | Pancreatic | Year of interview, education, tobacco smoking, history of diabetes and total energy intake |  |
| Bidoli et al. 2008 Italy | Dietary Intake | OR 1.20 (0.90,1.50) Trend:0.66 | Total SFA: Q2/Q1 | Renal Cell | Period of interview, education, tobacco smoking, alcohol drinking, treated hypertension, body mass index, family history of kidney cancer, and total energy intake |  |
| Jessri et al. 2011 Iran | Dietary Intake | OR 3.52 (1.10,3.89) Trend:0.01 | Total SFA: T3/T1 | Esophageal Squamous Cell Carcinoma | Age (years) and sex (male/female) |  |
| Bidoli et al. 2002 Iran | Dietary Intake | OR 0.80 (0.60,1.10) Trend:<0.05 | Total SFA: Q5/Q1 | Ovarian | Terms for age (5-year age categories), study center, year of interview, education, parity, oral contraceptive use, and energy intake |  |
| Polesel et al. 2007 Italy | Dietary Intake | OR 1.13 (0.58,2.21) Trend:0.79 | Total SFA: Q2/Q1 | Hepatocellular Carcinoma | Gender, age, centre, education, place of birth, hepatitis viruses (HBsAg+ and/or AntiHCV+ versus HBsAg– and AntiHCV–), drinking habits (Abstainers, Former, Current), maximal lifetime alcohol intake (P21 versus <21 drinks/week), and energy intake (kCal, energy from alcohol excluded). |  |
| Bassett et al. 2013 Australia | Blood | (1)HR 1.57 (1.10, 2.25) Trend:0.003 (2)HR 0.95 (0.68, 1.35) Trend:0.92 (3)HR 2.16 (1.46, 3.20) Trend:0.11 (4)HR 1.19 (0.83, 1.70) Trend:0.74 | (1)TotalSFA: Q4/Q1  (2)C14:0: Q5/Q1  (3)C16:0: Q3/Q1  (4)C18:0: Q4/Q1 | Prostate | Country of birth, education, alcohol intake, physical activity, total energy intake from food and family history of cancer and stratified by smoking status. |  |
|  | Dietary Intake | (1)HR 1.04 (0.72, 1.51) Trend:0.96 (2)HR 0.92 (0.65, 1.32) Trend:0.94 (3)HR 1.17 (0.82, 1.69) Trend:0.71 (4)HR 1.21 (0.85, 1.71) Trend:0.82 | (1)TotalSFA: Q4/Q1  (2)C14:0: Q4/Q1  (3)C16:0: Q5/Q1  (4)C18:0: Q2/Q1 |  |  |  |
| Pouchieu et al. 2014 UK | Blood | (1)OR 0.96 (0.58,1.61) Trend:0.1 (2)OR 1.27 (0.76,2.10) Trend:0.2 (3)OR 1.17 (0.70,1.95) Trend:0.1 (4)OR 1.05 (0.62,1.77) Trend:1.0 (5)OR 0.84 (0.48,1.49) Trend:0.8 | (1)TotalSFA: Q2/Q1  (2)C14:0: Q2/Q1  (3)C16:0: Q2/Q1  (4)C18:0: Q4/Q1 (5)C20:0: Q3/Q1 | Overall | Gender, age, intervention group (except in the models stratified on this variable), body mass index, height, smoking status, physical activity, alcohol intake, educational level and family history of cancer. |  |
| Wise et al. 2014 USA | Dietary Intake | (1)RR 1.01 (0.90, 1.13) Trend:0.02 (2)RR 1.00 (0.89, 1.13) Trend:0.009 (3)RR 0.96 (0.85, 1.08)  Trend:0.02 (4)RR 1.06 (0.94, 1.19) Trend:0.03 (5)RR 1.00 (0.89, 1.13) Trend:0.07 (6)RR 1.01 (0.90, 1.14) Trend:0.01 (7)RR 1.02 (0.90, 1.15) Trend:0.78 (8)RR 1.08 (0.95, 1.21) Trend:0.64 | (1)C4:0: Q2/Q1  (2)C6:0: Q2/Q1  (3)C8:0: Q2/Q1  (4)C10:0: Q2/Q1  (5)C12:0: Q3/Q1 (6)C14:0: Q2/Q1 (7)C16:0: Q4/Q1 (8)C18:0: Q4/Q1 | Uterine Leiomyomata | Age, questionnaire cycle, energy intake, age at menarche, parity, age at first birth, years since last birth, ever use of oral contraceptives, age at first oral contraceptive use, alcohol, smoking, BMI, education, occupation, income, marital status, and US region of residence. |  |
| Luu et al. 2018 China | Dietary Intake | HR 1.00 (0.85–1.17)  Trend:0.90 | Total SFA: Q5/Q1 | lung cancer | Age, ever smoking status, smoking packs-year (SMHS only), ever drinking status, BMI, physical activity status, vitamin supplemental use, menopausal status and hormone replacement therapy (SWHS only). |  |
| Kuriki et al. 2006 Japan | Blood | (1)OR 8.20 (2.86,23.52) Trend:<0.0001 (2)OR 1.66 (0.83,3.32) Trend:0.13 (3)OR 6.46 (2.41,17.26) Trend:<0.0005 (4)OR 0.84 (0.44,1.62) Trend:0.68 | (1)Total SFA: T3/T1  (2)C14:0: T3/T1  (3)C16:0: T3/T1  (4)C18:0: T3/T1 | Colorectal | BMI, habitual exercise, drinking and smoking status, green-yellow vegetable intake, and family history of colorectal cancer. |  |
| Sellem et al. 2018 French | Dietary Intake | HR 1.44 (1.10,1.87) Trend:0.008 | Total SFA: Q5/Q1 | Overall | Age (time-scale, years), gender, BMI (kg/m2), height (cm), physical activity (IPAQ categories: low, moderate, high or missing), smoking status (never smoked, former smoker, current smoker), number of 24-h dietary records, alcohol intake (g/days), energy intake without alcohol (kcal/d), family history of cancer (yes/no), educational level, total lipid intake (g/days), and fruit and vegetable intake (g/days, in models stratified for vitamin C) |  |
| Saadatian-Elahi et al. 2002 USA | Blood | (1)OR 1.75 (0.92,3.34) Trend:0.11 (2)OR 0.97 (0.52,1.81) Trend:0.86 (3)OR 1.64 (0.86,3.14) Trend:0.27 (4)OR 1.24 (0.66–2.36) Trend:0.79 | (1)Total SFA: Q3/Q1  (2)C14:0: Q3/Q1  (3)C16:0: Q4/Q1  (4)C18:0: Q2/Q1 | Breast | Age at first full-term birth, family history of breast cancer, history of benign breast disease, and total cholesterol. |  |
| Vinceti et al. 2013 Italy | Blood | (1)OR 1.66(0.39,7.16) Trend:0.646 (2)OR 3.00 (0.56,16.07) Trend:0.101 | (1)C17:0: Q3/Q1  (2)C18:0: Q3/Q1 | Cutaneous Melanoma | Phototype, education and sun exposure. |  |
| Nkondjock et al. 2005 Canada | Dietary Intake | (1)OR 0.83 (0.63,1.09) Trend:0.01 (2)OR 1.07 (0.81,1.42) Trend:0.62 (3)OR 0.99 (0.75,1.32) Trend:0.56 (4)OR 0.74 (0.56,0.97) Trend:0.02 (5)OR 0.82 (0.62,1.09) Trend:0.04 | (1)TotalSFA: Q2/Q1  (2)C4:0: Q2/Q1  (3)C12:0: Q2/Q1  (4)C16:0: Q3/Q1 (5)C18:0: Q3/Q1 | Pancreatic | Age, province, educational attainment, smoking, BMI, total fat and energy intake. |  |
