# Supplementary material for: Association of saturated fatty acids with cancer risk: a systematic review and meta-analysis
Source: Lipids Health Dis. 2024 Jan 30;23:32. doi: 10.1186/s12944-024-02025-z (PMC10826095; doi:10.1186/s12944-024-02025-z)
Supplement: Supplementary file 2 — Supplementary Material 2: Supplementary Table 2. The use of the Newcastle-Ottawa Scale for assessing the quality of the included articles. [file 12944_2024_2025_MOESM2_ESM.docx]

Supplementary Table 2 The use of the Newcastle-Ottawa Scale for assessing the quality of the included articles

1. **Case control study**

| Author | Year of Publication | Country | Selection of cases and controls | | | | Comparability | Exposure | | | Total score |
| --- | --- | --- | --- | --- | --- | --- | --- | --- | --- | --- | --- |
|  |  |  | Are the Case Definition and Diagnosis Adequate | Representativeness of the Cases | Selection of Controls | Definition of Controls |  | Ascertainment of Exposure | Are the Investigation Methods the Same for Cases and Controls | Non-Response Rate |  |
| Wu et al. | 2023 | China | 1 | 1 | 1 | 1 | 2 | 1 | 1 | 0 | 8 |
| Seyyedsalehi et al. | 2022 | Iran | 1 | 1 | 0 | 1 | 2 | 1 | 1 | 0 | 7 |
| Pan et al. | 2004 | Canada | 1 | 1 | 1 | 0 | 2 | 1 | 1 | 0 | 7 |
| Wakai et al. | 2000 | Japan | 1 | 1 | 0 | 1 | 2 | 1 | 1 | 0 | 7 |
| Do et al. | 2003 | Korean | 1 | 1 | 0 | 1 | 2 | 1 | 1 | 0 | 7 |
| Lucenteforte et al. | 2008 | Italy | 1 | 1 | 0 | 1 | 2 | 1 | 1 | 0 | 7 |
| Lucenteforte et al. | 2009 | Italy | 1 | 1 | 0 | 1 | 2 | 1 | 1 | 0 | 7 |
| Lucenteforte et al. | 2010 | Italy | 1 | 1 | 0 | 1 | 2 | 1 | 1 | 0 | 7 |
| Bidoli et al. | 2008 | Italy | 1 | 1 | 0 | 1 | 2 | 1 | 1 | 0 | 7 |
| Jessri et al. | 2011 | Iran | 1 | 1 | 0 | 1 | 2 | 1 | 1 | 0 | 7 |
| Bidoli et al. | 2002 | Iran | 1 | 1 | 0 | 1 | 2 | 1 | 1 | 0 | 7 |
| Polesel et al. | 2007 | Italy | 1 | 1 | 0 | 1 | 2 | 1 | 1 | 0 | 7 |
| Nkondjock et al. | 2003 | Canada | 1 | 1 | 1 | 0 | 2 | 1 | 1 | 0 | 7 |
| Mozafarinia et al. | 2021 | Iran | 1 | 1 | 1 | 0 | 2 | 1 | 1 | 0 | 7 |
| Fan et al. | 2022 | China | 1 | 1 | 0 | 1 | 2 | 1 | 1 | 0 | 7 |
| Tu et al. | 2022 | China | 1 | 1 | 1 | 1 | 2 | 0 | 1 | 0 | 7 |
| Chun et al. | 2015 | Korea | 1 | 1 | 1 | 0 | 2 | 1 | 1 | 0 | 7 |
| Jackson et al. | 2012 | Jamaica | 1 | 1 | 0 | 1 | 2 | 1 | 1 | 0 | 7 |
| Nkondjock et al. | 2003 | Canada | 1 | 1 | 1 | 0 | 2 | 1 | 1 | 0 | 7 |
| Matta et al. | 2022 | USA | 0 | 1 | 1 | 1 | 2 | 1 | 1 | 0 | 7 |
| Matejcic et al. | 2018 | Denmark、France、Greece、Germany、Italy、Netherlands、Norway、Spain、Sweden、UK | 1 | 1 | 1 | 0 | 2 | 1 | 1 | 0 | 7 |
| Vlajinac et al. | 1997 | Serbia | 1 | 1 | 0 | 1 | 2 | 1 | 1 | 0 | 7 |
| Zhu et al. | 2019 | China | 1 | 1 | 1 | 0 | 2 | 1 | 1 | 0 | 7 |
| Shannon et al. | 2007 | China | 1 | 1 | 1 | 0 | 2 | 1 | 1 | 0 | 7 |
| Hirko et al. | 2018 | USA | 0 | 1 | 1 | 1 | 2 | 1 | 1 | 0 | 7 |
| Bravi et al. | 2013 | Italy and Switzerland | 1 | 1 | 0 | 1 | 2 | 1 | 1 | 0 | 7 |
| Challier et al. | 1998 | French | 1 | 1 | 1 | 0 | 2 | 1 | 1 | 0 | 7 |
| Pouchieu et al. | 2014 | UK | 1 | 1 | 1 | 0 | 2 | 1 | 1 | 0 | 7 |
| Kuriki et al. | 2006 | Japan | 1 | 1 | 0 | 1 | 2 | 1 | 1 | 0 | 7 |
| Saadatian-Elahi et al. | 2002 | USA | 1 | 1 | 1 | 0 | 2 | 1 | 1 | 0 | 7 |
| Vinceti et al. | 2013 | Italy | 1 | 1 | 1 | 0 | 2 | 1 | 1 | 0 | 7 |
| Nkondjock et al. | 2005 | Canada | 1 | 1 | 1 | 0 | 2 | 1 | 1 | 0 | 7 |
| Takata et al. | 2009 | USA | 1 | 1 | 1 | 1 | 2 | 1 | 1 | 0 | 8 |
| Chavarro et al. | 2013 | USA | 1 | 1 | 1 | 1 | 2 | 1 | 1 | 0 | 8 |
| Shishavan et al. | 2020 | Iran | 1 | 1 | 1 | 1 | 2 | 1 | 1 | 0 | 8 |
| Crowe et al. | 2008 | Denmark,Germany, Greece, Italy, Netherlands, Spain, Sweden, and UK | 1 | 1 | 1 | 1 | 2 | 1 | 1 | 0 | 8 |
| Gong et al. | 2010 | USA | 1 | 1 | 1 | 0 | 2 | 1 | 1 | 1 | 8 |
| Bassett et al. | 2013 | Australia | 1 | 1 | 1 | 1 | 2 | 1 | 1 | 0 | 8 |

**b. Cohort study**

| Author | Year of Publication | Country | Selection of cohorts | | | | Comparability | Outcome | | | Total score | Follow-up duration |
| --- | --- | --- | --- | --- | --- | --- | --- | --- | --- | --- | --- | --- |
|  |  |  | Representativeness of the Exposed Cohort | Selection of the Non-Exposed Cohort | Ascertainment of Exposure | Demonstration That Outcome of Interest Was Not Present at Start of Study | Comparability of the Exposed Cohort and the Non-Exposed Cohort | Assessment of Outcome | Was Follow-Up Long Enough for Outcomes to Occur | Adequacy of Follow Up |  |  |
| Knekt et al. | 1990 | Finland | 1 | 1 | 1 | 1 | 2 | 1 | 1 | 1 | 9 | 20 |
| Voorrips et al. | 2002 | Netherlands | 1 | 1 | 1 | 1 | 2 | 1 | 1 | 1 | 9 | 6.3 |
| Wakai et al. | 2005 | Japan | 1 | 1 | 0 | 1 | 2 | 1 | 1 | 1 | 8 | 7.6±1.8 |
| Lof et al. | 2007 | Sweden | 1 | 1 | 0 | 1 | 2 | 1 | 1 | 1 | 8 | 13 |
| Kurahashi et al. | 2008 | Japan | 1 | 1 | 0 | 1 | 2 | 1 | 1 | 1 | 8 | 7.5 |
| Thiébaut et al. | 2009 | USA | 1 | 1 | 1 | 1 | 2 | 1 | 1 | 1 | 9 | 6.3±1.2 |
| Gilsing et al. | 2011 | Netherlands | 1 | 1 | 0 | 1 | 2 | 1 | 1 | 1 | 8 | 16.3 |
| Sczaniecka et al. | 2012 | USA | 1 | 1 | 0 | 1 | 2 | 1 | 1 | 1 | 8 | 6 |
| Wise et al. | 2014 | USA | 1 | 1 | 0 | 1 | 2 | 1 | 1 | 0 | 7 | 8 |
| Hodge et al. | 2015 | Italian and Greek | 1 | 1 | 0 | 1 | 2 | 1 | 1 | 1 | 8 | 9 |
| Kraja et al. | 2015 | Netherlands | 1 | 1 | 1 | 1 | 2 | 1 | 1 | 1 | 9 | 14.6 |
| Luu et al. | 2018 | China | 1 | 1 | 1 | 1 | 2 | 1 | 1 | 1 | 9 | mean±SD in SWHS: 8.3±4.0 years for cases vs 13.9±1.8 years for noncases; and SMSH: 4.6±2.6 years for cases vs 8.4±1.5 years for noncases; p < 0.0001 in both cohorts |
| Sellem et al. | 2018 | French | 1 | 1 | 0 | 1 | 2 | 1 | 1 | 1 | 8 | 7.6 |
| Cai et al. | 2020 | Japan | 1 | 1 | 1 | 1 | 2 | 1 | 1 | 1 | 9 | median follow-up period, 16.0 years |
| Aglago et al. | 2021 | Denmark、France、Greece、Germany、Italy、Netherlands、Norway、Spain、Sweden、UK | 1 | 1 | 1 | 1 | 2 | 1 | 1 | 1 | 9 | median follow-up = 15 years |
| Shishavan et al. | 2021 | Iran | 1 | 1 | 1 | 1 | 2 | 1 | 1 | 1 | 9 | 4 |
| Shimomura et al. | 2022 | Japan | 1 | 1 | 0 | 1 | 2 | 1 | 1 | 0 | 7 | 13-17 |
