# Supplementary material for: Association of saturated fatty acids with cancer risk: a systematic review and meta-analysis
Source: Lipids Health Dis. 2024 Jan 30;23:32. doi: 10.1186/s12944-024-02025-z (PMC10826095; doi:10.1186/s12944-024-02025-z)
Supplement: Supplementary file 4 — Supplementary Material 4: Supplementary File 2. Search history. [file 12944_2024_2025_MOESM4_ESM.docx]

Supplementary File 2 Search history

1.PubMed

| Search number | Query | Sort By | Filters | Search Details | Results | Time |
| --- | --- | --- | --- | --- | --- | --- |
| 17 | ((("Fatty Acids"[Mesh]) OR (((((((Fatty Acids[Title/Abstract]) OR (Fatty Acid[Title/Abstract])) OR (Saturated Fatty Acids[Title/Abstract])) OR (Saturated Fatty Acid[Title/Abstract])) OR (Aliphatic Acids[Title/Abstract])) OR (Aliphatic Acid[Title/Abstract])) OR (FFA[Title/Abstract]))) AND (("Neoplasms"[Mesh]) OR (((((((((((Neoplasms[Title/Abstract]) OR (Tumor[Title/Abstract])) OR (Neoplasm[Title/Abstract])) OR (Tumors[Title/Abstract])) OR (Neoplasia[Title/Abstract])) OR (Neoplasias[Title/Abstract])) OR (Cancer[Title/Abstract])) OR (Cancers[Title/Abstract])) OR (Malignancy[Title/Abstract])) OR (Malignancies[Title/Abstract])) OR (malignant neoplasm[Title/Abstract])))) AND (((("Cohort Studies"[Mesh]) OR (Cohort)) OR (("Case-Control Studies"[Mesh]) OR (Case Control))) OR (("Cross-Sectional Studies"[Mesh]) OR (Cross Sectional))) |  |  | ("Fatty Acids"[MeSH Terms] OR ("Fatty Acids"[Title/Abstract] OR "fatty acid"[Title/Abstract] OR "saturated fatty acids"[Title/Abstract] OR "saturated fatty acid"[Title/Abstract] OR "aliphatic acids"[Title/Abstract] OR "aliphatic acid"[Title/Abstract] OR "FFA"[Title/Abstract])) AND ("Neoplasms"[MeSH Terms] OR ("Neoplasms"[Title/Abstract] OR "Tumor"[Title/Abstract] OR "Neoplasm"[Title/Abstract] OR "Tumors"[Title/Abstract] OR "Neoplasia"[Title/Abstract] OR "Neoplasias"[Title/Abstract] OR "Cancer"[Title/Abstract] OR "Cancers"[Title/Abstract] OR "Malignancy"[Title/Abstract] OR "Malignancies"[Title/Abstract] OR "malignant neoplasm"[Title/Abstract])) AND ("Cohort Studies"[MeSH Terms] OR ("cohort"[All Fields] OR "cohort s"[All Fields] OR "cohorte"[All Fields] OR "cohorts"[All Fields]) OR ("Case-Control Studies"[MeSH Terms] OR (("ieee int conf automation sci eng case"[Journal] OR "case phila"[Journal] OR "case"[All Fields]) AND ("controling"[All Fields] OR "controllability"[All Fields] OR "controllable"[All Fields] OR "controllably"[All Fields] OR "controller"[All Fields] OR "controller s"[All Fields] OR "controllers"[All Fields] OR "controlling"[All Fields] OR "controls"[All Fields] OR "prevention and control"[MeSH Subheading] OR ("prevention"[All Fields] AND "control"[All Fields]) OR "prevention and control"[All Fields] OR "control"[All Fields] OR "control groups"[MeSH Terms] OR ("control"[All Fields] AND "groups"[All Fields]) OR "control groups"[All Fields]))) OR ("Cross-Sectional Studies"[MeSH Terms] OR (("cross"[All Fields] OR "crosse"[All Fields] OR "crossed"[All Fields] OR "crosses"[All Fields] OR "crossing"[All Fields] OR "crossings"[All Fields]) AND ("sectional"[All Fields] OR "sectionally"[All Fields] OR "sectionals"[All Fields])))) | 4,289 | 11:35:31 |
| 16 | ((("Cohort Studies"[Mesh]) OR (Cohort)) OR (("Case-Control Studies"[Mesh]) OR (Case Control))) OR (("Cross-Sectional Studies"[Mesh]) OR (Cross Sectional)) |  |  | "Cohort Studies"[MeSH Terms] OR ("cohort"[All Fields] OR "cohort s"[All Fields] OR "cohorte"[All Fields] OR "cohorts"[All Fields]) OR ("Case-Control Studies"[MeSH Terms] OR (("ieee int conf automation sci eng case"[Journal] OR "case phila"[Journal] OR "case"[All Fields]) AND ("controling"[All Fields] OR "controllability"[All Fields] OR "controllable"[All Fields] OR "controllably"[All Fields] OR "controller"[All Fields] OR "controller s"[All Fields] OR "controllers"[All Fields] OR "controlling"[All Fields] OR "controls"[All Fields] OR "prevention and control"[MeSH Subheading] OR ("prevention"[All Fields] AND "control"[All Fields]) OR "prevention and control"[All Fields] OR "control"[All Fields] OR "control groups"[MeSH Terms] OR ("control"[All Fields] AND "groups"[All Fields]) OR "control groups"[All Fields]))) OR ("Cross-Sectional Studies"[MeSH Terms] OR (("cross"[All Fields] OR "crosse"[All Fields] OR "crossed"[All Fields] OR "crosses"[All Fields] OR "crossing"[All Fields] OR "crossings"[All Fields]) AND ("sectional"[All Fields] OR "sectionally"[All Fields] OR "sectionals"[All Fields]))) | 3,997,970 | 11:35:11 |
| 15 | ("Cross-Sectional Studies"[Mesh]) OR (Cross Sectional) |  |  | "Cross-Sectional Studies"[MeSH Terms] OR (("cross"[All Fields] OR "crosse"[All Fields] OR "crossed"[All Fields] OR "crosses"[All Fields] OR "crossing"[All Fields] OR "crossings"[All Fields]) AND ("sectional"[All Fields] OR "sectionally"[All Fields] OR "sectionals"[All Fields])) | 679,093 | 11:34:50 |
| 14 | Cross Sectional |  |  | ("cross"[All Fields] OR "crosse"[All Fields] OR "crossed"[All Fields] OR "crosses"[All Fields] OR "crossing"[All Fields] OR "crossings"[All Fields]) AND ("sectional"[All Fields] OR "sectionally"[All Fields] OR "sectionals"[All Fields]) | 543,380 | 11:34:42 |
| 13 | "Cross-Sectional Studies"[Mesh] | Most Recent |  | "Cross-Sectional Studies"[MeSH Terms] | 484,964 | 11:34:35 |
| 12 | ("Case-Control Studies"[Mesh]) OR (Case Control) |  |  | "Case-Control Studies"[MeSH Terms] OR (("ieee int conf automation sci eng case"[Journal] OR "case phila"[Journal] OR "case"[All Fields]) AND ("controling"[All Fields] OR "controllability"[All Fields] OR "controllable"[All Fields] OR "controllably"[All Fields] OR "controller"[All Fields] OR "controller s"[All Fields] OR "controllers"[All Fields] OR "controlling"[All Fields] OR "controls"[All Fields] OR "prevention and control"[MeSH Subheading] OR ("prevention"[All Fields] AND "control"[All Fields]) OR "prevention and control"[All Fields] OR "control"[All Fields] OR "control groups"[MeSH Terms] OR ("control"[All Fields] AND "groups"[All Fields]) OR "control groups"[All Fields])) | 1,792,861 | 11:34:20 |
| 11 | Case Control |  |  | ("ieee int conf automation sci eng case"[Journal] OR "case phila"[Journal] OR "case"[All Fields]) AND ("controling"[All Fields] OR "controllability"[All Fields] OR "controllable"[All Fields] OR "controllably"[All Fields] OR "controller"[All Fields] OR "controller s"[All Fields] OR "controllers"[All Fields] OR "controlling"[All Fields] OR "controls"[All Fields] OR "prevention and control"[MeSH Subheading] OR ("prevention"[All Fields] AND "control"[All Fields]) OR "prevention and control"[All Fields] OR "control"[All Fields] OR "control groups"[MeSH Terms] OR ("control"[All Fields] AND "groups"[All Fields]) OR "control groups"[All Fields]) | 464,906 | 11:34:11 |
| 10 | "Case-Control Studies"[Mesh] | Most Recent |  | "Case-Control Studies"[MeSH Terms] | 1,462,493 | 11:34:02 |
| 9 | ("Cohort Studies"[Mesh]) OR (Cohort) |  |  | "Cohort Studies"[MeSH Terms] OR "cohort"[All Fields] OR "cohort s"[All Fields] OR "cohorte"[All Fields] OR "cohorts"[All Fields] | 2,944,403 | 11:33:54 |
| 8 | Cohort |  |  | "cohort"[All Fields] OR "cohort s"[All Fields] OR "cohorte"[All Fields] OR "cohorts"[All Fields] | 1,007,586 | 11:33:46 |
| 7 | "Cohort Studies"[Mesh] | Most Recent |  | "Cohort Studies"[MeSH Terms] | 2,545,959 | 11:33:38 |
| 6 | ("Neoplasms"[Mesh]) OR (((((((((((Neoplasms[Title/Abstract]) OR (Tumor[Title/Abstract])) OR (Neoplasm[Title/Abstract])) OR (Tumors[Title/Abstract])) OR (Neoplasia[Title/Abstract])) OR (Neoplasias[Title/Abstract])) OR (Cancer[Title/Abstract])) OR (Cancers[Title/Abstract])) OR (Malignancy[Title/Abstract])) OR (Malignancies[Title/Abstract])) OR (malignant neoplasm[Title/Abstract])) |  |  | "Neoplasms"[MeSH Terms] OR "Neoplasms"[Title/Abstract] OR "Tumor"[Title/Abstract] OR "Neoplasm"[Title/Abstract] OR "Tumors"[Title/Abstract] OR "Neoplasia"[Title/Abstract] OR "Neoplasias"[Title/Abstract] OR "Cancer"[Title/Abstract] OR "Cancers"[Title/Abstract] OR "Malignancy"[Title/Abstract] OR "Malignancies"[Title/Abstract] OR "malignant neoplasm"[Title/Abstract] | 4,984,212 | 11:33:10 |
| 5 | ((((((((((Neoplasms[Title/Abstract]) OR (Tumor[Title/Abstract])) OR (Neoplasm[Title/Abstract])) OR (Tumors[Title/Abstract])) OR (Neoplasia[Title/Abstract])) OR (Neoplasias[Title/Abstract])) OR (Cancer[Title/Abstract])) OR (Cancers[Title/Abstract])) OR (Malignancy[Title/Abstract])) OR (Malignancies[Title/Abstract])) OR (malignant neoplasm[Title/Abstract]) |  |  | "Neoplasms"[Title/Abstract] OR "Tumor"[Title/Abstract] OR "Neoplasm"[Title/Abstract] OR "Tumors"[Title/Abstract] OR "Neoplasia"[Title/Abstract] OR "Neoplasias"[Title/Abstract] OR "Cancer"[Title/Abstract] OR "Cancers"[Title/Abstract] OR "Malignancy"[Title/Abstract] OR "Malignancies"[Title/Abstract] OR "malignant neoplasm"[Title/Abstract] | 3,573,703 | 11:33:02 |
| 4 | "Neoplasms"[Mesh] | Most Recent |  | "Neoplasms"[MeSH Terms] | 3,903,895 | 11:32:55 |
| 3 | ("Fatty Acids"[Mesh]) OR (((((((Fatty Acids[Title/Abstract]) OR (Fatty Acid[Title/Abstract])) OR (Saturated Fatty Acids[Title/Abstract])) OR (Saturated Fatty Acid[Title/Abstract])) OR (Aliphatic Acids[Title/Abstract])) OR (Aliphatic Acid[Title/Abstract])) OR (FFA[Title/Abstract])) |  |  | "Fatty Acids"[MeSH Terms] OR "Fatty Acids"[Title/Abstract] OR "fatty acid"[Title/Abstract] OR "saturated fatty acids"[Title/Abstract] OR "saturated fatty acid"[Title/Abstract] OR "aliphatic acids"[Title/Abstract] OR "aliphatic acid"[Title/Abstract] OR "FFA"[Title/Abstract] | 647,717 | 11:32:46 |
| 2 | ((((((Fatty Acids[Title/Abstract]) OR (Fatty Acid[Title/Abstract])) OR (Saturated Fatty Acids[Title/Abstract])) OR (Saturated Fatty Acid[Title/Abstract])) OR (Aliphatic Acids[Title/Abstract])) OR (Aliphatic Acid[Title/Abstract])) OR (FFA[Title/Abstract]) |  |  | "fatty acids"[Title/Abstract] OR "fatty acid"[Title/Abstract] OR "saturated fatty acids"[Title/Abstract] OR "saturated fatty acid"[Title/Abstract] OR "aliphatic acids"[Title/Abstract] OR "aliphatic acid"[Title/Abstract] OR "FFA"[Title/Abstract] | 647,717 | 11:32:36 |
| 1 | "Fatty Acids"[Mesh] | Most Recent |  | "Fatty Acids"[MeSH Terms] | 500,151 | 11:32:26 |

2.Cochrane Search

Search Name:

Date Run: 6/12/2023 17:15:03

Comment:

ID Search Hits

#1 MeSH descriptor: [Fatty Acids] explode all trees 26141

#2 (Fatty Acids or Fatty Acid or Saturated Fatty Acids or Saturated Fatty Acid or Aliphatic Acids or Aliphatic Acid or FFA):ti,ab,kw 22199

#3 #1 or #2 39995

#4 MeSH descriptor: [Neoplasms] explode all trees 113676

#5 (Neoplasms or Tumor or Neoplasm or Tumors or Neoplasia or Neoplasias or Cancer or Cancers or Malignancy or Malignancies  or malignant neoplasm):ti,ab,kw 258015

#6 #4 or #5 271516

#7 MeSH descriptor: [Cohort Studies] explode all trees 184770

#8 (cohort) 73958

#9 #7 or #8 239970

#10 MeSH descriptor: [Case-Control Studies] explode all trees 23326

#11 (case control) 45598

#12 #10 or #11 61055

#13 MeSH descriptor: [Cross-Sectional Studies] explode all trees 8329

#14 (cross sectional) 21120

#15 #13 or #14 21120

#16 #9 or #12 or #15 291352

#17 #3 and #6 and #16 697

3.Embase

| No. | Query | Results | Date |
| --- | --- | --- | --- |
| #17 | #3 AND #6 AND #16 | 5184 | 6-Dec-23 |
| #16 | #9 OR #12 OR #15 | 2625730 | 6-Dec-23 |
| #15 | #13 OR #14 | 838242 | 6-Dec-23 |
| #14 | 'cross sectional' | 838242 | 6-Dec-23 |
| #13 | 'cross-sectional study'/exp | 595782 | 6-Dec-23 |
| #12 | #10 OR #11 | 295421 | 6-Dec-23 |
| #11 | 'case control' | 295421 | 6-Dec-23 |
| #10 | 'case control study'/exp | 227261 | 6-Dec-23 |
| #9 | #7 OR #8 | 1647181 | 6-Dec-23 |
| #8 | cohort | 1647181 | 6-Dec-23 |
| #7 | 'cohort analysis'/exp | 1082715 | 6-Dec-23 |
| #6 | #4 OR #5 | 7046653 | 6-Dec-23 |
| #5 | 'neoplasms':ab,ti OR 'neoplasm':ab,ti OR 'tumors':ab,ti OR 'tumor':ab,ti OR 'neoplasias':ab,ti OR 'neoplasia':ab,ti OR 'cancers':ab,ti OR 'cancer':ab,ti OR 'malignancies':ab,ti OR 'malignancy':ab,ti OR '?malignant neoplasm':ab,ti | 4821192 | 6-Dec-23 |
| #4 | 'malignant neoplasm'/exp OR 'neoplasm'/exp | 6146871 | 6-Dec-23 |
| #3 | #1 OR #2 | 878861 | 6-Dec-23 |
| #2 | 'fatty acids':ab,ti OR 'fatty acid':ab,ti OR 'saturated fatty acids':ab,ti OR 'saturated fatty acid':ab,ti OR 'aliphatic acids':ab,ti OR 'aliphatic acid':ab,ti OR ffa:ab,ti | 320362 | 6-Dec-23 |
| #1 | 'fatty acid'/exp | 774643 | 6-Dec-23 |

4.Web of science

| Entitlements | # | Search Query | Database | Results | Date Run |
| --- | --- | --- | --- | --- | --- |
| - WOS: 1900 to 2023 - BIOSIS: 1994 to 2023 - CSCD: 1989 to 2023 - DIIDW: 1966 to 2023 - KJD: 1980 to 2023 - MEDLINE: 1950 to 2023 - SCIELO: 2002 to 2023 | 1 | Fatty Acids (Topic) OR Fatty Acid (Topic) OR Saturated Fatty Acids (Topic) OR Saturated Fatty Acid (Topic) OR Aliphatic Acids (Topic) OR Aliphatic Acid (Topic) OR FFA (Topic) | All Databases | 465303 | Wed Dec 6 2023 17:30:58 GMT+0800 (China Standard Time) |
| - WOS: 1900 to 2023 - BIOSIS: 1994 to 2023 - CSCD: 1989 to 2023 - DIIDW: 1966 to 2023 - KJD: 1980 to 2023 - MEDLINE: 1950 to 2023 - SCIELO: 2002 to 2023 | 2 | Neoplasms (Title) OR Tumor (Title) OR Neoplasm (Title) OR Tumors (Title) OR Neoplasia (Title) OR Neoplasias (Title) OR Cancer (Title) OR Cancers (Title) OR Malignancy (Title) OR Malignancies (Title) OR malignant neoplasm (Title) | All Databases | 2304556 | Wed Dec 6 2023 17:30:58 GMT+0800 (China Standard Time) |
| - WOS: 1900 to 2023 - BIOSIS: 1994 to 2023 - CSCD: 1989 to 2023 - DIIDW: 1966 to 2023 - KJD: 1980 to 2023 - MEDLINE: 1950 to 2023 - SCIELO: 2002 to 2023 | 3 | cohort (Topic) OR case control (Topic) OR cross sectional (Topic) | All Databases | 2321170 | Wed Dec 6 2023 17:30:58 GMT+0800 (China Standard Time) |
| - WOS: 1900 to 2023 - BIOSIS: 1994 to 2023 - CSCD: 1989 to 2023 - DIIDW: 1966 to 2023 - KJD: 1980 to 2023 - MEDLINE: 1950 to 2022 - SCIELO: 2002 to 2022 | 4 | #1 AND #2 AND #3 | All Databases | 1172 | Wed Dec 6 2023 :30:58 GMT+0800 (China Standard Time) |
