# Supplementary material for: Association of saturated fatty acids with cancer risk: a systematic review and meta-analysis
Source: Lipids Health Dis. 2024 Jan 30;23:32. doi: 10.1186/s12944-024-02025-z (PMC10826095; doi:10.1186/s12944-024-02025-z)

Supplementary File 3 Meta-analysis for the SFA subtypes and cancer

1.Meta-analysis for the SFA subtypes and cancer :C4:0、C6:0、C8:0、C10:0


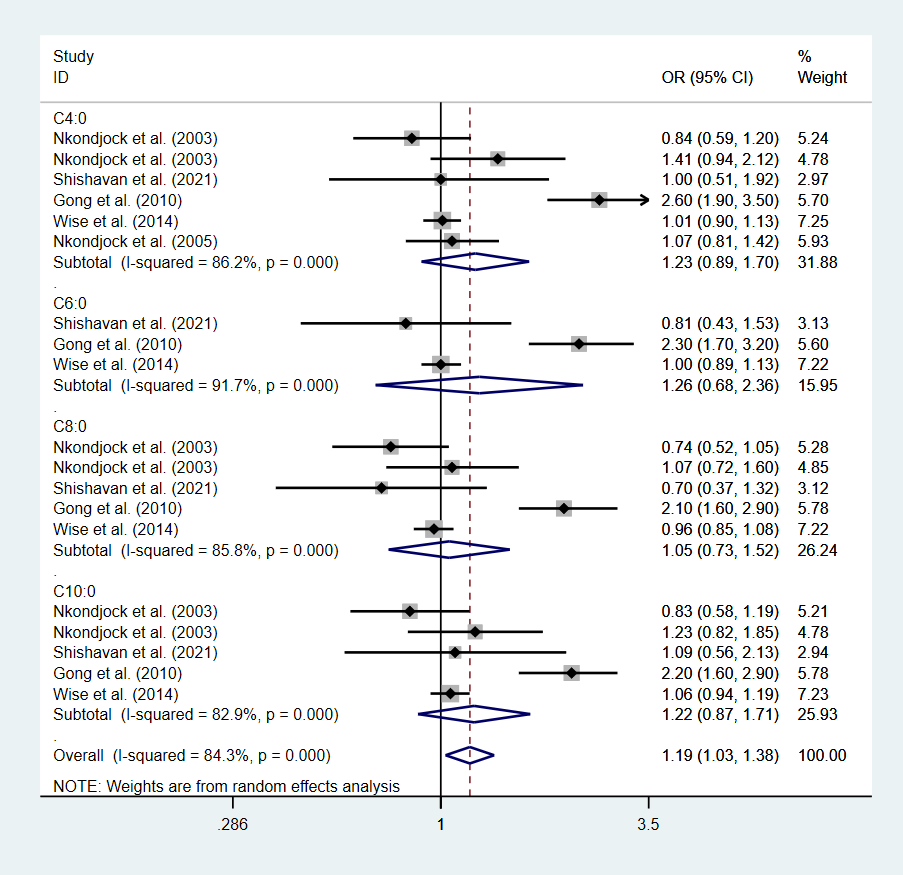


2.Meta-analysis for C12:0 and cancer


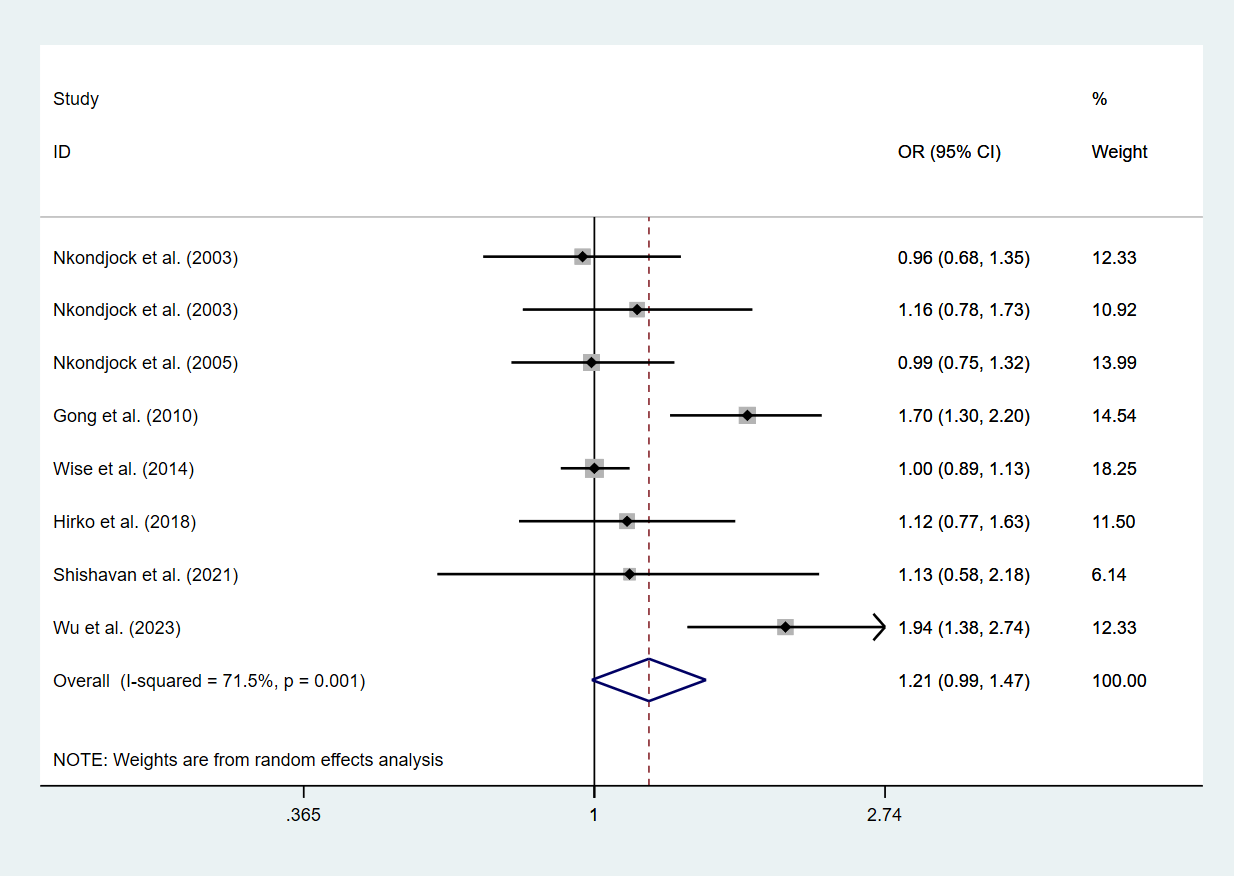


3.Meta-analysis for C14:0 and cancer


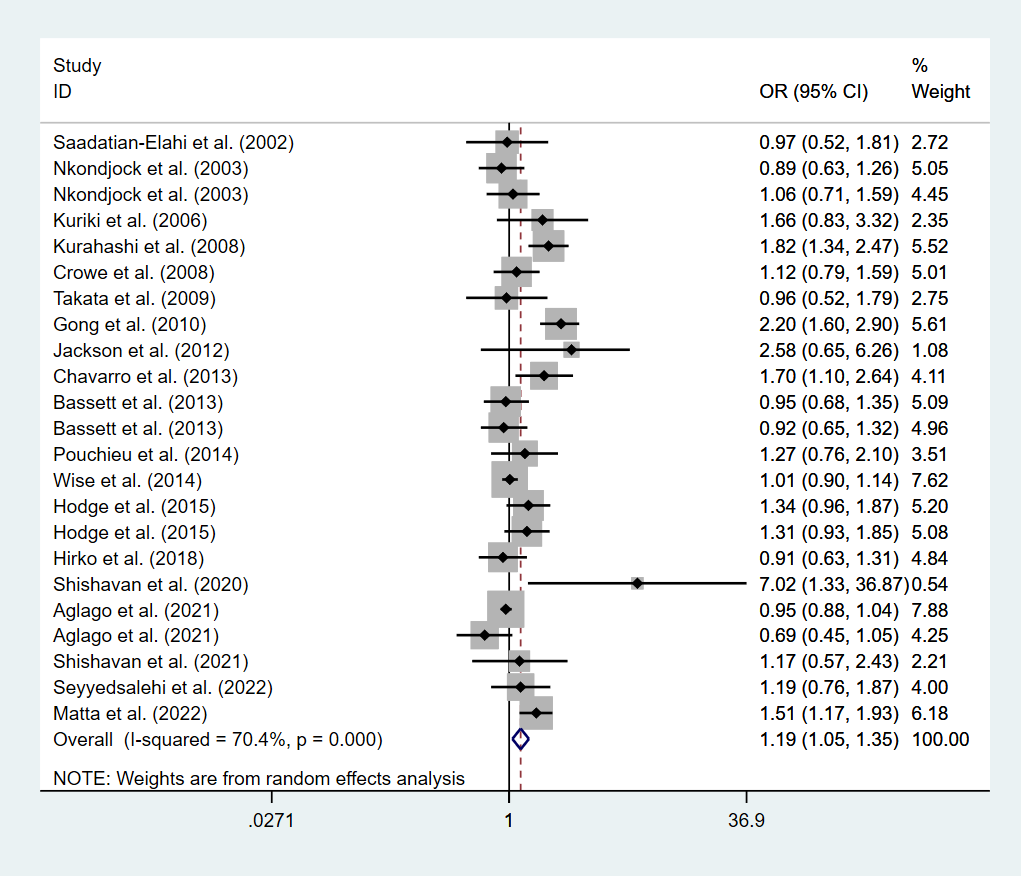


4.Meta-analysis for C15:0 and cancer


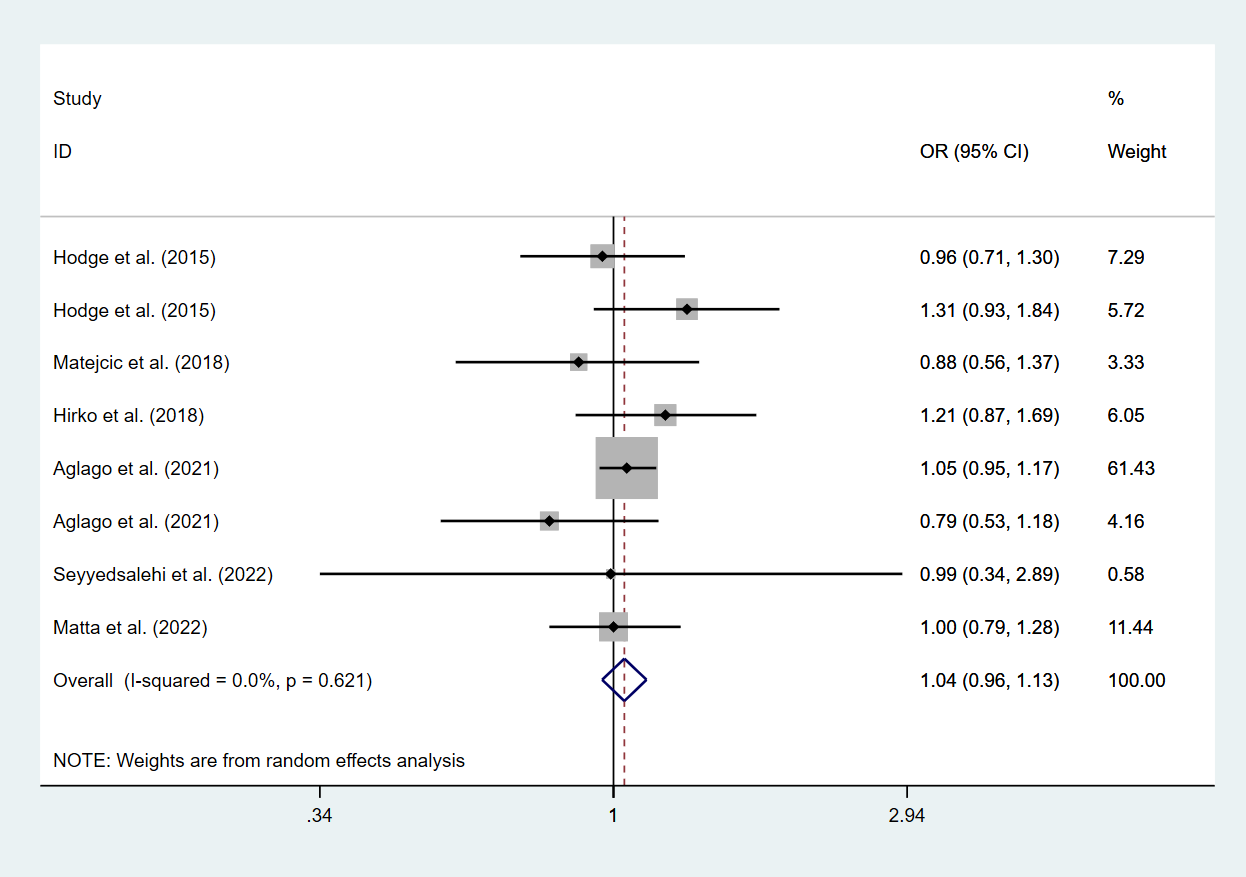


5.Meta-analysis for C16:0 and cancer


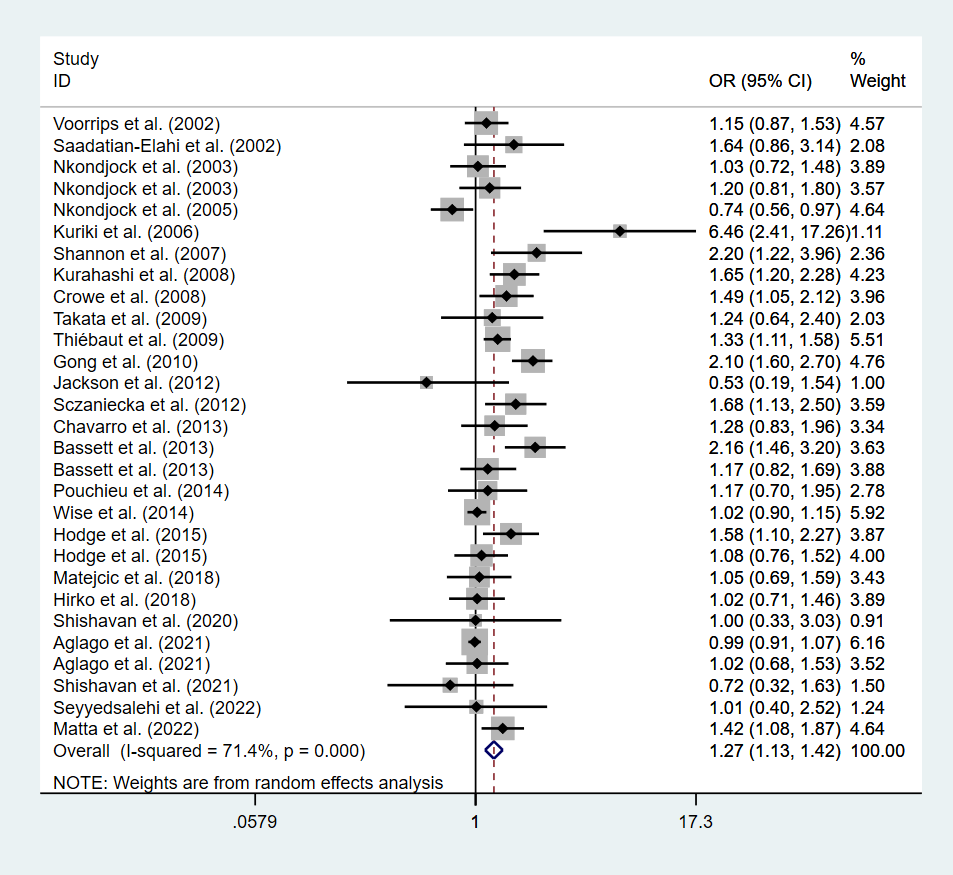


6.Meta-analysis for C17:0 and cancer


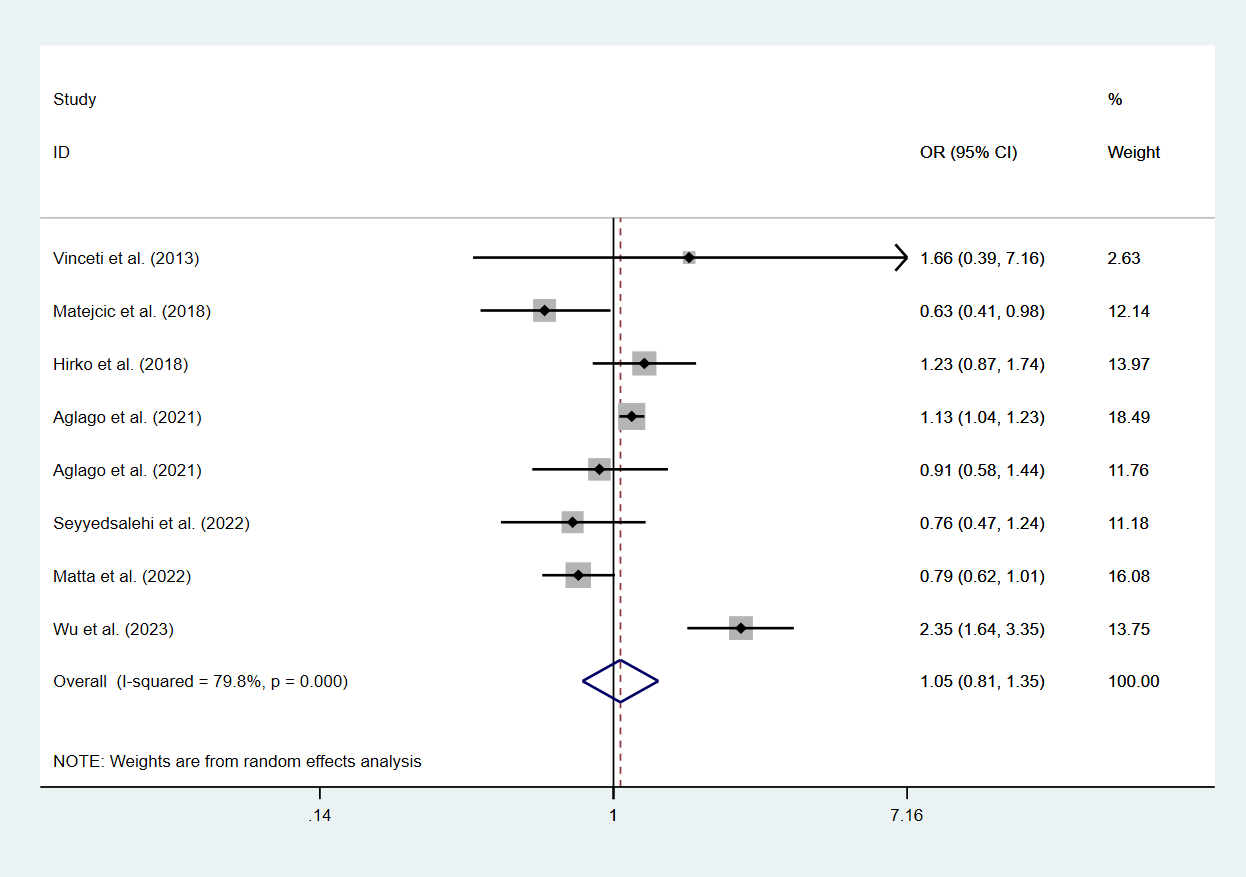


7.Meta-analysis for C18:0 and cancer


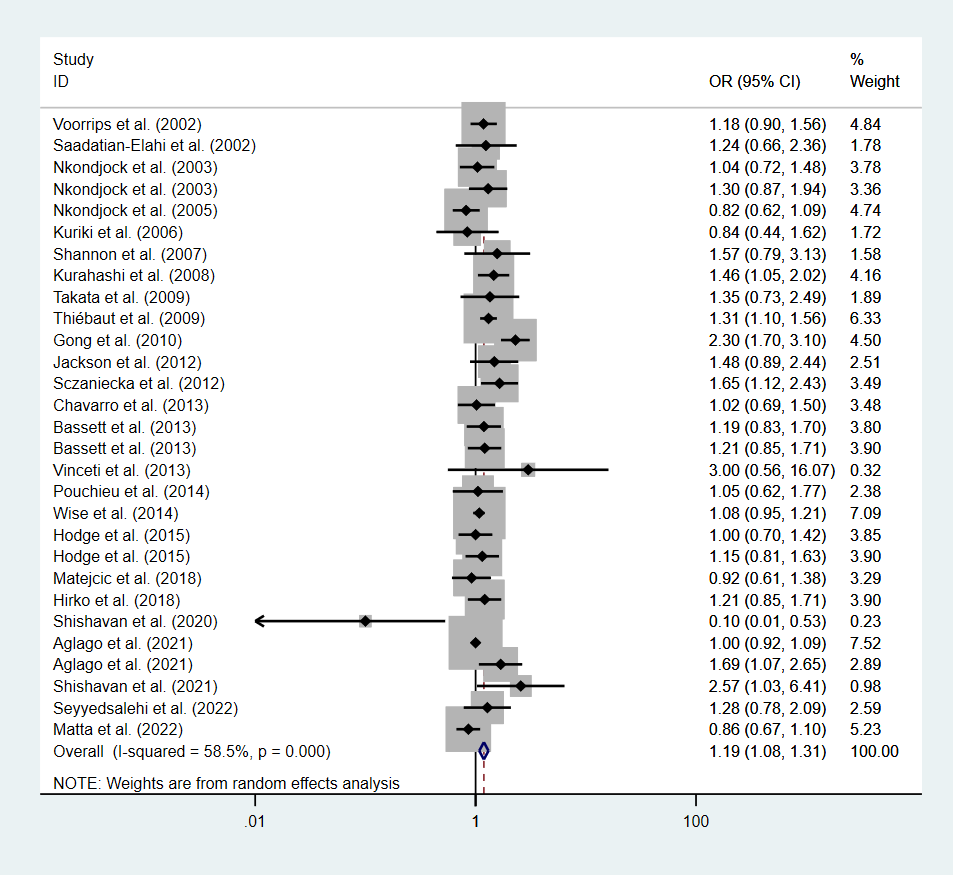


1. Meta-analysis for SFA subtypes and cancer :C20:0、C22:0


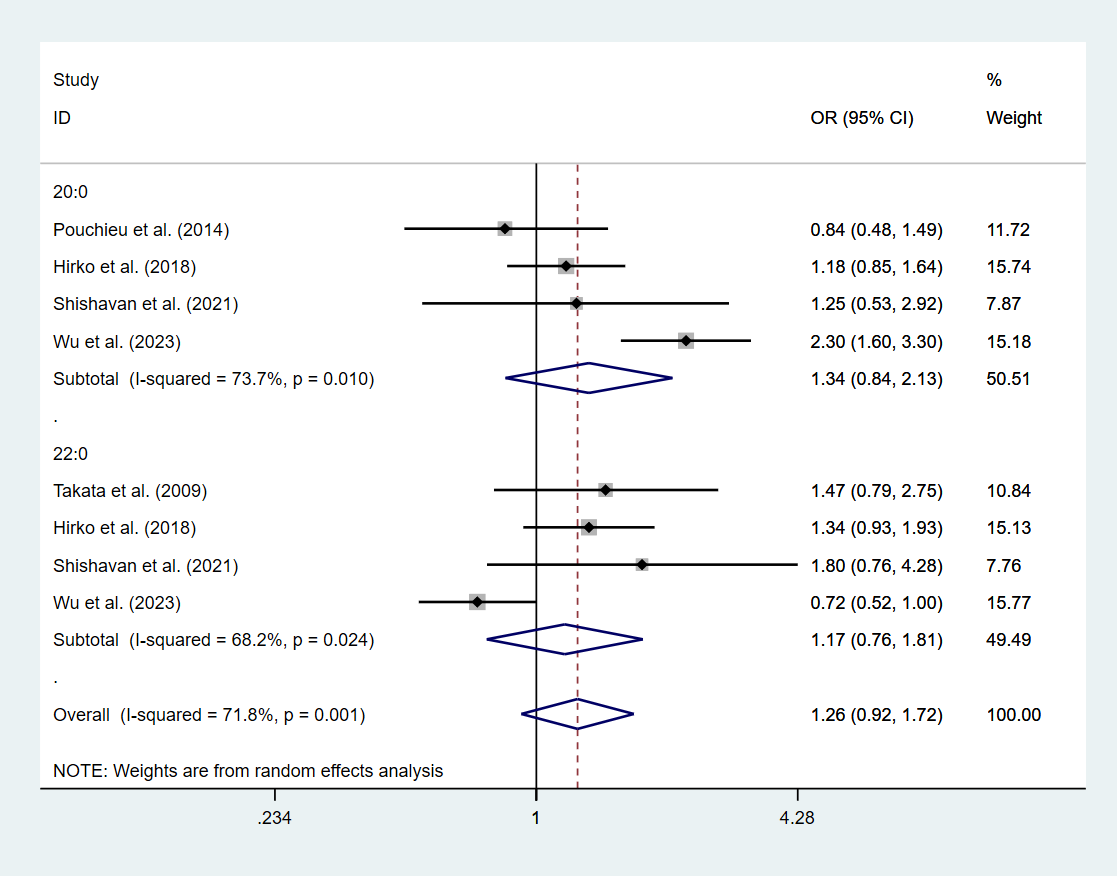


9.Meta-analysis for C24:0 and cancer


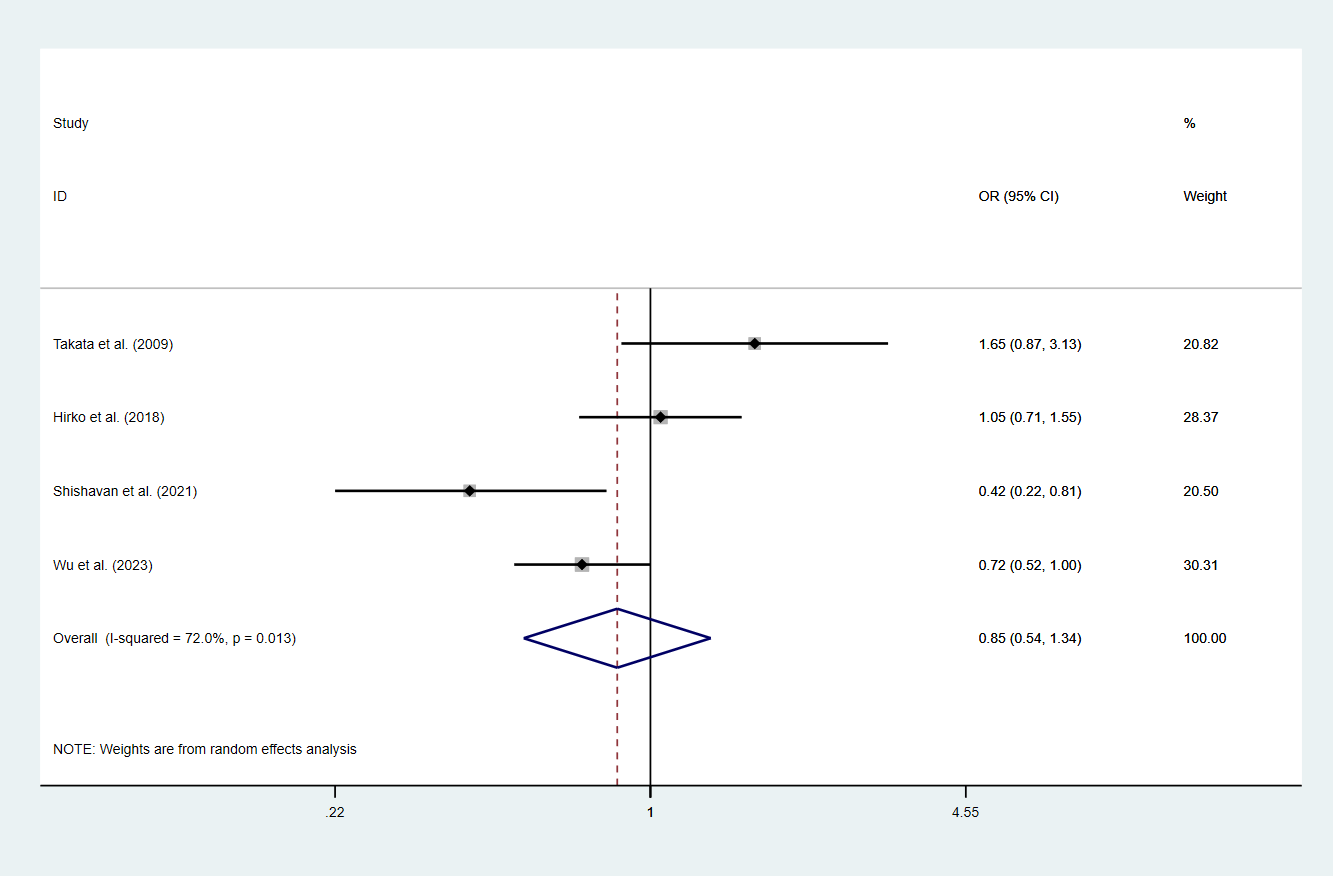

Supplement: Supplementary file 5 — Supplementary Material 5: Supplementary File 3. Meta-analysis for the SFA subtypes and cancer. [file 12944_2024_2025_MOESM5_ESM.docx]
