# Supplementary material for: Association of saturated fatty acids with cancer risk: a systematic review and meta-analysis
Source: Lipids Health Dis. 2024 Jan 30;23:32. doi: 10.1186/s12944-024-02025-z (PMC10826095; doi:10.1186/s12944-024-02025-z)

Supplementary File 4 Additional analyses

1.Begg’s test

2.Egger’s test

3.Sensitivity analysis

4.Publication bias data


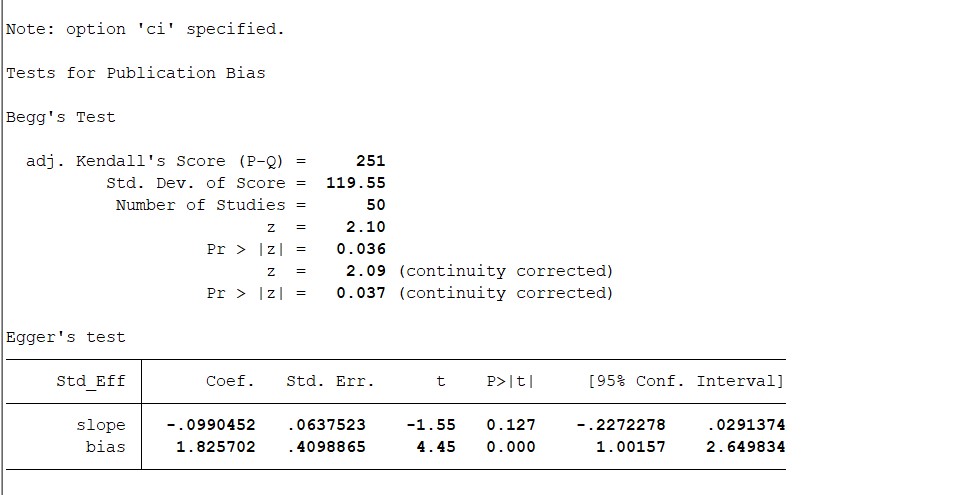

Supplement: Supplementary file 6 — Supplementary Material 6: Supplementary File 4. Additional analyses: Begg’s test; Egger’s test; Sensitivity analysis; Publication bias evaluation. [file 12944_2024_2025_MOESM6_ESM.docx]
